# Synching with seasonality: Predicting roe deer parturition phenology across its distributional range

**DOI:** 10.1111/1365-2656.70148

**Published:** 2025-10-03

**Authors:** Johanna Kauffert, A. J. Mark Hewison, Benedikt Gehr, Torsten Hothorn, Sophie Baur, Jean‐Michel Gaillard, Petter Kjellander, Andreas König, Jörg Müller, Manuela Panzacchi, Maryline Pellerin, Balint Tamasi, Wibke Peters, Annette Menzel

**Affiliations:** ^1^ Professorship of Ecoclimatology, TUM School of Life Sciences Technical University of Munich Freising Germany; ^2^ Université de Toulouse, INRAE, CEFS Castanet‐Tolosan France; ^3^ LTSER ZA PYRénées GARonne Auzeville Tolosane France; ^4^ Department of Evolutionary Biology and Environmental Studies University of Zurich Zurich Switzerland; ^5^ Wildtier Schweiz Zürich Switzerland; ^6^ Epidemiology, Biostatistics and Prevention Institute University of Zurich Zurich Switzerland; ^7^ Bavarian State Institute of Forestry, Research Unit Wildlife Biology and Management Freising Germany; ^8^ Unité Mixte de Recherche 5558 ‘Biométrie et Biologie Evolutive’, CNRS‐Université Lyon 1 Bâtiment Mendel Villeurbanne Cedex France; ^9^ Grimsö Wildlife Research Station, Department of Ecology Swedish University of Agricultural Sciences, SLU Riddarhyttan Sweden; ^10^ Wildlife Biology and Management Unit TUM School of Life Sciences, Technical University of Munich Freising Germany; ^11^ Professorship for Conservation Biology and Forest Ecology Julius‐Maximilians University Würzburg Rauhenebrach Germany; ^12^ Bavarian Forest National Park Grafenau Germany; ^13^ Norwegian Institute for Nature Research Trondheim Norway; ^14^ Office Français de la Biodiversité, Direction de la Recherche et de l'Appui Scientifiques Service Conservation et Gestion Durable des Espèces Exploitées Chateauvillain France; ^15^ Swiss Tropical and Public Health Institute Allschwil Switzerland; ^16^ University of Basel Basel Switzerland; ^17^ Institute for Advanced Study, Technical University of Munich Garching Germany

**Keywords:** distributional regression models, environmental predictability, GAMLSS, life‐history events, parturition phenology, roe deer

## Abstract

1. Latitude and elevation are the most commonly studied drivers of large‐scale variation in the phenology of life‐history events. However, these coarse gradients cannot reliably predict observed spatial variation in phenology. Although it is less often investigated, environmental predictability is also a selective force that constrains spatial variation in life‐history events.
2. Here, we explore how environmental predictability contributes to shaping spatial variation in the parturition phenology of roe deer across its distributional range in Europe. We compiled data on roe deer parturition dates across Europe within the research collaboration EURODEER, and from citizen scientists and related birth dates to elevation and environmental predictability, measured by Colwell's metrics of contingency and constancy, based on high‐resolution climate and NDVI values. We predicted parturition timing and synchrony simultaneously within a single modelling framework using a distributional regression model (i.e. GAMLSS).
3. Our approach provided more robust predictions of variation in birth phenology than commonly used approaches based on the combination of latitude and elevation. We found that roe deer align their parturition dates with both elevation and seasonality in environmental conditions. We also identified an apparent shift towards later parturition from west to east across the distributional range in Europe, putatively linked to relatively milder and more constant climates in the west. Contrary to our expectations, we did not find any consistent link between parturition synchrony and environmental predictability, suggesting that other factors, such as small‐scale heterogeneities in landscape composition, play a key role.
4. Our work emphasizes the importance of understanding macrophenological processes in the variation of life‐history event timing across space. It also highlights the need to account for this spatial variation when investigating region‐specific adaptations, particularly in light of climate change.

## Graphical abstract

Using an unprecedentedly large data set on roe deer parturition dates, our study shows that elevation and Colwell's metrics of environmental predictability enhance the prediction of the timing of parturition across Europe, compared to using latitude and elevation alone. Through advanced modelling, we identified a shift towards later parturition from west to east.

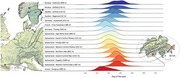

## 1 INTRODUCTION

Life‐history events are influenced by environmental cues and their predictability (Marshall & Burgess, 2015; Visser et al., 2010). Species have evolved life‐history tactics that enable them to perceive, adjust to and anticipate alterations in their environment through variations in, for example, photoperiodism, temperature or precipitation (Bernhardt et al., 2020; Forrest & Miller‐Rushing, 2010; Helm et al., 2013). Due to the Earth's axial tilt, several of these seasonal changes, such as solar radiation, photoperiod and air temperature, vary annually along latitudes, directly affecting the phenology and primary production of terrestrial vegetation and creating moderately predictable seasons (Hut et al., 2013). Consequently, the immutable annual cycle of photoperiod is often used by birds and mammals as a proximate cue to time life‐history events to the lagged changes in the ultimate driver of green‐up, that is, temperature (annual hysteresis, Helm et al., 2013; Hut et al., 2013; Immelmann, 1972). Thus, proxy variables such as latitude and elevation are frequently used in studies of spatial variation in the timing of life‐history events to account for environmental heterogeneity (cf. the use of Hopkins Bioclimatic law in Peláez et al. (2020)). However, latitude is often a poor predictor of fine‐scale variation in vegetation green‐up due to local climatic conditions. These deviations have already been observed to influence the timing of species' life‐history events, which appear to be more strongly driven by plant phenology in relation to local climatic conditions (Stoner et al., 2016). Elevation, in contrast, was found to be a strong predictor of life‐history timing, likely due to its pronounced effects on local climatic and phenological conditions over short spatial scales (e.g. Bears et al., 2009; Peláez et al., 2020; Perfito et al., 2004). However, as Peláez et al. (2020) also showed, in situations where microclimatic anomalies disrupt the expected altitudinal gradients in temperature and phenology, elevation may fail to accurately predict parturition timing. Hence, the combination of latitudinal and altitudinal gradients may not fully explain the timing of life‐history events (Linnell & Andersen, 1998; Peláez et al., 2020) and, hence, may not provide a reliable basis for strong biological inference in macroecological studies. Here, we evaluate whether environmental predictability and elevation together better explain variation in roe deer (*Capreolus capreolus* L.) parturition phenology compared to the combination of latitude and elevation.

**FIGURE 1:**
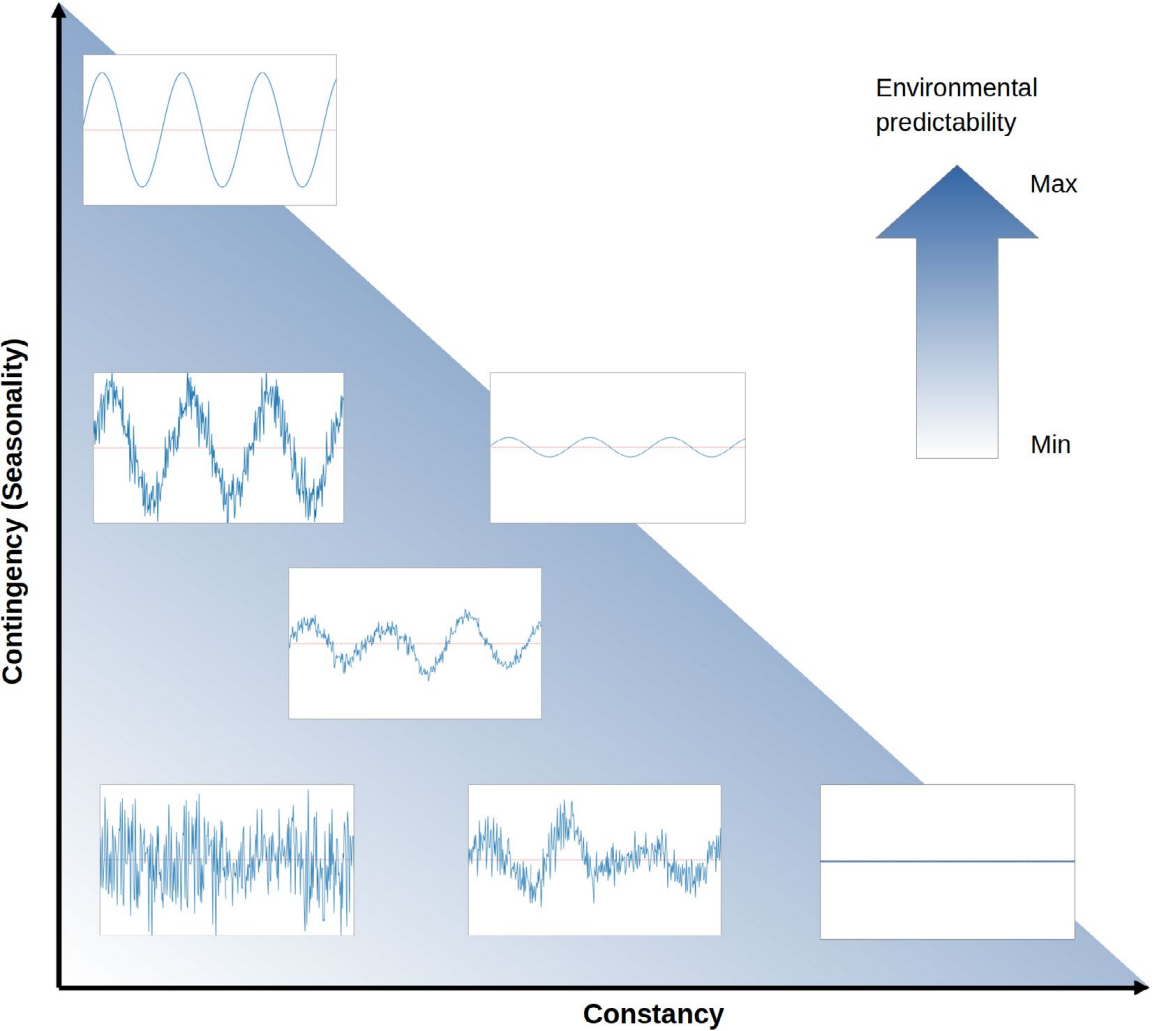
Colwell's (1974) metric of predictability based on the interplay between constancy and contingency (adapted from Riotte‐Lambert and Matthiopoulos (2020)).

Environmental predictability and seasonality are distinct selective forces that markedly influence life‐history evolution (Marshall & Burgess, 2015; Tonkin et al., 2017; Tuljapurkar et al., 2009; Varpe, 2017), enabling species to synchronize periods of higher energy demands with peaks in resource availability (McNamara & Houston, 2008; Post & Forchhammer, 2008; Visser et al., 1998). Thus, habitats with high environmental predictability provide organisms with a degree of certainty to anticipate the environmental conditions and time their trait expression accordingly (Bernhardt et al., 2020; Reed et al., 2010). For example, the timing of breeding is a crucial life‐history event, with both short‐ and long‐term effects on individual fitness, requiring close alignment with high resource availability (Clutton‐Brock et al., 1987; Plard et al., 2015; Plard, Gaillard, Coulson, Hewison, Delorme, Warnant, & Bonenfant, 2014). A variety of approaches have been proposed to index environmental predictability and seasonality, often based on the temporal fluctuations of environmental variables. However, measures of seasonality, typically approximated by a given metric measured over a predefined time frame, may primarily reflect amplitude while failing to account for the repeatability inherent in these observed temporal patterns, so that important aspects of the driving forces may be overlooked (Bitter et al., 2021; Tonkin et al., 2017). In this context, Colwell (1974) introduced a series of metrics to measure environmental predictability by assessing temporal patterns of physical or biological fluctuations. According to these metrics, complete environmental predictability is achieved when an environmental variable is perfectly constant (*constancy metric*) or perfectly periodic (*contingency metric*) within a defined area and time frame (Figure 1; Colwell, 1974; Riotte‐Lambert & Matthiopoulos, 2020).

Relating Colwell's metrics of predictability to the breeding phenology of mammals has received little attention so far, with only two studies focusing on ungulates (English et al., 2012; Loe et al., 2005). Ungulates are particularly well suited for studying the relationship between environmental predictability and breeding phenology as they inhabit a wide range of climatic and geographically diverse habitats (Lovari et al., 2016; Whitehead, 1972) and exhibit correspondingly highly variable breeding phenology (English et al., 2012). Even within a given species, variation in breeding phenology is generally large across the distributional range (Loe et al., 2005; Peláez et al., 2020; Rehnus et al., 2020).

In temperate regions, ungulates must align the energy‐demanding periods of late gestation and lactation with the concurrent green‐up of vegetation in spring, making optimal parturition timing indispensable (Gaillard et al., 2013; Plard, Gaillard, Coulson, Hewison, Delorme, Warnant, & Bonenfant, 2014; Rivrud et al., 2016). This is especially crucial for income breeders (sensu Jönsson, 1997) that must finance reproduction from current intake rather than stored reserves. Late‐born individuals often suffer negative fitness consequences due to insufficient development time before winter onset (Feder et al., 2008), while early‐born individuals are at higher risk of dying of hypothermia (Linnell & Andersen, 1998). In response to this strong selection pressure to match reproductive allocation with the peak of high‐forage availability, parturition synchrony is expected to be strongest when the vegetation growing season is short and predictable (Rutberg, 1987). However, due to unprecedented changes in weather fluctuations and in their habitats caused by anthropogenic climate change, animals run an increasing risk of experiencing trophic mismatches between their reproductive phenology and the availability of necessary resources to finance allocation (Radchuk et al., 2019; Visser & Gienapp, 2019). For example, in the face of the ever‐increasing advance of spring phenology that results from global warming (Menzel et al., 2020; Menzel, Sparks, et al., 2006), animals should shift their reproductive timing accordingly to continue tracking optimal rearing conditions. Alternatively, herbivores may increasingly shift their home ranges across diverse habitats to experience a prolonged window of peak forage availability (Albon & Langvatn, 1992). For example, in agricultural landscapes, roe deer populations experience small‐scale heterogeneity in phenological peaks that differ in cultivated fields compared to remnant woodland patches (Abbas et al., 2011), with an apparent impact on birth timing and synchrony (Brunot et al., 2025). Despite ongoing research, our understanding of how climatic and plant phenological properties across different regions and ecological gradients impact the timing and synchrony of life‐history events, such as breeding, remains limited (Chmura et al., 2019; English et al., 2012; Loe et al., 2005). Evaluating life‐history variation and its drivers over a species' entire distributional range is essential and could help predict future shifts (Bailey et al., 2022; Thackeray et al., 2016). Here, we aim to test whether components of environmental predictability related to weather and plant phenological conditions, together with elevation, a widely established proxy for local climatic and phenological conditions, provide a more robust prediction of spatial variation in the timing of parturition than the usual combination of latitude and elevation.

To do that, we analyse an exceptional data set on roe deer, the most widespread and abundant ungulate in Central Europe (Apollonio et al., 2010; Lister et al., 1998), to assess female parturition phenology in relation to environmental predictability across the species distributional range in Europe (Figure S1 in the Appendix). Roe deer are of particular interest in this regard because their parturition phenology has been reported to vary considerably within their distributional range (Linnell & Andersen, 1998; Peláez et al., 2020). Furthermore, roe deer are closer to the ‘income’ end of the capital‐income breeder continuum (Jönsson, 1997; see supplementary table in Andersen et al., 2000 for evidence); hence, there should be a particularly strong imperative for females to align the energy‐demanding periods of late gestation and lactation with the availability of high‐quality forage (Gaillard et al., 2013; Plard, Gaillard, Coulson, Hewison, Delorme, Warnant, & Bonenfant, 2014). Additionally, roe deer are unique among ungulates in that they exhibit an extended period of embryonic diapause (Aitken, 1974), potentially providing a flexible mechanism to adjust birth timing independently of mating phenology (Kauffert, Ehrmantraut, et al., 2025; Renfree & Fenelon, 2017), although phenotypic plasticity in birth date has been shown to be limited (Plard et al., 2013). We hypothesized that variation in birth phenology of roe deer is better explained by also considering environmental predictability, measured by Colwell's metrics of constancy and contingency, together with elevation, rather than by latitude and elevation alone. While latitude and elevation serve as generic proxies for environmental variation over continuous gradients, using climatic and plant phenological data (Schultz & Halpert, 1993) with Colwell's metrics, which capture the predictable components of their variation, provides potentially more informative predictors. Further, we predicted mean parturition timing and parturition synchrony over the roe deer's distributional range across Europe. Due to the longer time window with favourable weather conditions for plant growth in regions with higher environmental constancy and at lower elevations, we expected a pronounced response in terms of earlier parturition. Second, we expected to observe the highest parturition synchrony in regions with particularly high levels of contingency and at higher elevations in response to strong selection pressure to match increased energetic requirements with the short and sharp window of high‐forage availability (Loe et al., 2005; Rutberg, 1987).

## 2 MATERIALS AND METHODS

### 2.1 Study regions and parturition data

**FIGURE 2:**
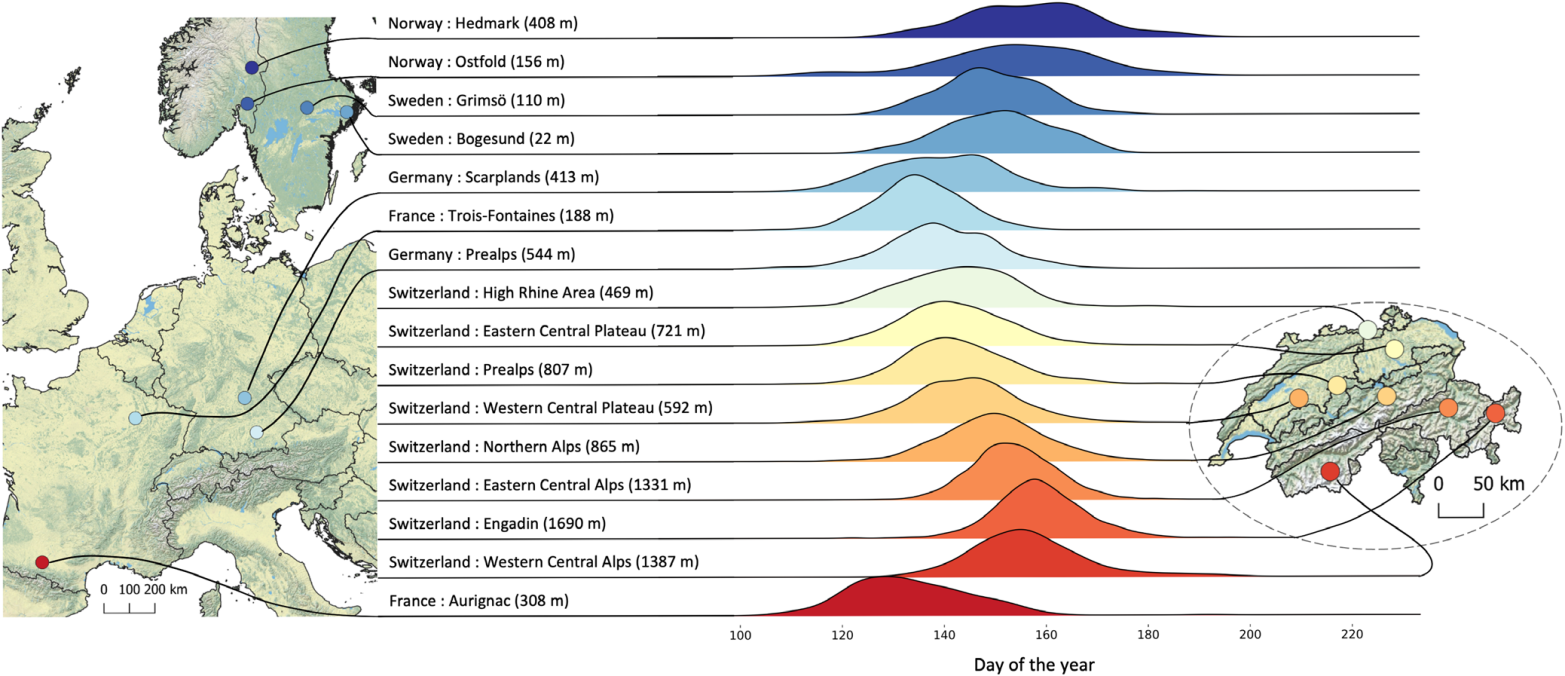
Distribution of parturition dates (DoY—day of year) for roe deer fawns across the different study regions in Europe with the median elevation of all birth sites per region given in brackets in m a.s.l. sorted by latitude (represented by a heat‐colour scale, from red at the lowest latitudes to blue at the highest latitudes). For further information on sampling details, such as the number of observations and the observation period, refer to Table S1 in the Appendix.

Parturition dates of fawns (*n* = 17,210) originated from citizen scientists and different research groups, which were consolidated within a Pan‐European project within the EURODEER Collaborative Project (Urbano & Cagnacci, 2021). We obtained fawn parturition dates from 16 study sites across Europe (Figure 2, see also Table S1 in the Appendix) representing five countries: Norway (Panzacchi, 2007; approval by the Norwegian animal ethics committee: 2004/48647), Sweden (Davis et al., 2016; approvals by the Ethical Committee of Animal Experiments in Uppsala, Sweden: C289/2009, C302/2012, C149/2015, 5.8.18–22,179–2021), Germany (Kauffert et al., 2023; no ethical approval required), France (Hewison et al., 2009; Plard, Gaillard, Coulson, Hewison, Delorme, Warnant, & Bonenfant, 2014; approvals by the French Ministry for Research and Higher Education and the ethics committee: Apafis#39320, Apafis#7880, Apafis#42152‐2023021016306261 v3) and Switzerland (Peláez et al., 2020; Rehnus & Reimoser, 2014; no ethical approval required).

### 2.2 Environmental data

We retrieved the elevation at each birth site from ALOS World 3D‐30m (AW3D30) (Takaku et al., 2020), as this source provides consistent coverage at all latitudes. As a proxy of forage availability for large herbivores, we calculated the Normalized Difference Vegetation Index (NDVI) using satellite remote sensing data (Pettorelli et al., 2005, 2011), specifically MODIS (Terra Surface Reflectance 8‐Day Global 250 m, MOD09Q1) within a circular buffer of 500 m radius around the birth site. We employed a smoothed time series of NDVI (2001–2020) using the Savitzky‐Golay filter (window length = 45 days, poly‐order = 2) (Chen et al., 2004; Savitzky & Golay, 1964) to retrieve mean monthly NDVI values. MODIS and AW3D30 data were accessed via Python's Google Earth Engine API (Gorelick et al., 2017). We retrieved total daily precipitation (mm) and daily mean temperature (°C) data from the E‐OBS Gridded Dataset (v26.0) with a spatial resolution of 0.1 degree in regular latitude/longitude coordinates (Cornes et al., 2018). For each fawn location, we calculated contingency (i.e. the repeatability of periodicity of a given variable across years, which takes values between 0 and 1, where 1 indicates perfect periodicity; this metric is often interpreted as indicative of the degree of seasonality) and constancy (i.e. the level of intra‐annual variability, which takes values between 0 and 1, with 1 indicating perfect constancy across seasons; for formula see Colwell, 1974) for three environmental drivers: NDVI, precipitation and mean temperature, based on monthly averaged NDVI and weekly total precipitation and weekly averaged temperature time series from 2001 to 2020 covering the 12 months of the year. To predict the parturition phenology of roe deer across its distributional range in Europe, we set up a 50 km equally spaced point grid covering the extent of the E‐OBS data set (excluding Northern Africa, West Asia (except Türkiye) and large water bodies) and calculated these metrics for the entire point grid across Europe.

### 2.3 Statistical analysis

Parturition dates were available for a range of landscapes, varying in geographical extent, elevation, sampling periods and sampling techniques (see corresponding studies). Notably, the Swiss data were collected mainly by hunters within the project ‘Rehkitzmarkierung Schweiz’ (Rehnus & Reimoser, 2014), which is not limited to a specific region but spans the whole of this topographically diverse country (highest reported parturition location at 2700 m). Therefore, we divided the data in relation to Switzerland's bio‐geographical regions (Gonseth et al., 2001). Data collection in Switzerland was carried out by hunters and was partly motivated by mitigating fawn mortality due to mowing activities, which may potentially introduce a degree of sampling bias (Kauffert et al., 2023). Hence, we first investigated whether this sampling protocol generated an apparent trend towards earlier parturition dates over time resulting from a shift to an earlier start of searches for fawns in response to ‘false’ phenological events (Menzel, von Vopelius, et al., 2006; Schnelle, 1955). ‘False’ phenological events, such as mowing and hay‐cutting, are not only a response to plant phenology but are closely dependent on human agricultural practices. For this, we used quantile regression for each region to determine whether the 0.1, 0.5 and 0.9 quantiles of the day of fawn marking against the year revealed statistically significant trends (*α* = 0.05) towards earlier searches over the study period (see Supporting Information). As a result, we excluded two sub‐regions (Jura and Swiss South Alps) from the final data set where data collection was progressively earlier over time.

Age estimation of fawns is challenging and can only be undertaken by trained staff within a few days after birth, unless the mother doe is repeatedly observed during the period around parturition (Jullien et al., 1992; Rehnus et al., 2018; Stamm et al., 2017). Therefore, back‐calculated parturition dates of fawns carry a degree of uncertainty that increases with the age of the fawn. Hence, we modelled parturition date as an interval‐censored variable, with an increasing interval range corresponding to the fawn's estimated age (age: <7 days: ±2; age: 8–14 days ±3; age: >15 days ±5) (Kauffert et al., 2023). We specified the interval‐censored dependent variable as a survival object (Surv()) with the survival package (Therneau et al., 2023). We used Generalized Additive Models for Location, Scale and Shape (GAMLSS) to model parturition timing and synchrony simultaneously within a single modelling framework (gamlss package; 10.32614/CRAN.package.gamlss; Rigby et al., 2005) in R 4.3.1. Compared to modelling synchrony as an aggregated dependent variable that leads to potential loss of information and heterogeneity, we were able to take advantage of all dependent data points. The location‐scale regression model describes the influence of the explanatory variables on the location parameter (corresponding to a ‘mean’) and the scale parameter (i.e. dispersion corresponding to a ‘standard deviation’). We used the interval‐censored Normal family (NOic) to model roe deer parturition dates by a normal distribution (Gaillard et al., 1993). Within this distribution, the location parameter was modelled with an identity link function and can be understood as mean parturition timing, *μ*. The scale parameter *σ* describing parturition synchrony was modelled using a log link (Rigby et al., 2019). To facilitate comparison of the scale parameter with previous studies, we report the predictions for synchrony as the 80% confidence interval. Hence, in our analysis, location and scale may vary across regions, years and environmental variables, resulting in not only different means, but also different variances potentially explained by the environmental variables.

We fitted three models: (1) The ‘Null Model’, describing parturition timing and synchrony in relation to region and elevation (two fixed effects); (2) The ‘Latitude‐Elevation Model’ including the effects of elevation and latitude (two fixed effects), as described by Peláez et al. (2020); and (3) The ‘Colwell‐Elevation Model’ including the effects of contingency and constancy for NDVI, precipitation and temperature, in addition to elevation (seven fixed effects in total). For comparison, we additionally fitted a model including only Colwell's metrics (‘Colwell‐only Model’) (six fixed effects; see Appendix S1). For each of our models, we included a random effect for the interaction between region (*n* = 16) and the respective year to account for region‐specific annual fluctuations in parturition dates, for example, due to variable sampling effort (Kauffert et al., 2023). First, we predicted parturition date for three different elevation classes per region that corresponded to the 10% lowest, the 10% around the median and the 10% highest parturition locations in the data set. Next, we predicted parturition date using the ‘Latitude‐Elevation Model’ and the ‘Colwell‐Elevation Model’ for each grid point within the distributional range of roe deer in Europe. All predictions were computed conditionally with respect to mean parameter estimates for the random effects.

To check the assumption of normality for the birth date distribution, we used a Shift‐Scale Mixed Effect Transformation Model with an interval‐censored dependent variable with no explicit distributional assumptions (Hothorn et al., 2018; Siegfried et al., 2023; Tamási, 2025) in the tramME R package (10.32614/CRAN.package.tramME; Tamasi & Hothorn, 2023). In this model class, the dependent variable is conditionally transformed to a reference distribution with a covariate‐dependent flexible transformation function (Hothorn et al., 2018). Hence, this model class does not make any a priori assumptions about the data distribution.

### 2.4 Model validation

We used published empirical distribution parameters for parturition date across Europe (reviewed in Linnell & Andersen, 1998; Peláez et al., 2020) to validate our model (Table 1). None of these data were analysed in the previous steps of our approach; hence, providing a completely independent validation. We excluded study regions with *n* <= 30 and when only the date of marking was provided, with no estimation for the fawn's age to ensure reliable parameter estimation (Linnell & Andersen, 1998). Validating the model against independent empirical data offers a more rigorous test of its generalizability and ecological relevance than within‐sample comparison alone (Yates et al., 2023).

**TABLE 1:** Reported empirical distributions of parturition dates across Europe (from Linnell & Andersen, 1998; Peláez et al., 2020). The mean parturition date was not specifically reported for Poland (Kałuzinski, 1982).

Sample region	Period	*n*	Mean/synchrony median (80%)		Prediction ‘Latitude‐Elevation Model’				Prediction ‘Colwell‐Elevation Model’				References
					Mean	Diff.	Synch.	Diff.	Mean	Diff.	Synch.	Diff.	
England, Chedington	1968–1972	42	131	18	144	13	24	6	127	−4	27	9	Gill (1994)
Germany, Baden‐Wuerttemberg	1973–2019	16,130	141	22	145	4	24	2	145	4	23	1	Hagen et al. (2021)
Poland, Czempiń	1976–1980	129	153		144	−9	24		145	−8	25		Kałuzinski (1982), Peláez et al. (2020)
Italy, Tyrol	1983–1992	113	164	21	154	−10	22	1	156	−8	21	0	Linnell and Andersen (1998), Wotschikowsky and Schwab (1994)
Sweden, Ekenäs	1986–1999	233	153	25	149	‐4	24	−1	145	−8	25	0	Jarnemo et al. (2004)
Norway, Storfosna	1991–1994	296	142	26	155	13	24	−2	141	−1	23	−3	Linnell and Andersen (1998)
Norway, Jøa	1992–1993	45	142	28	153	11	25	−3	142	0	22	−6	Linnell and Andersen (1998)
Italy, Apennines	1997–2003	117	150	17	143	−7	23	6	148	−2	23	6	Raganella‐Pelliccioni et al. (2007)
Spain, Madrid			139		140	1	23		137	−2	28		Plard (2014)

## 3 RESULTS

### 3.1 Environmental predictability

**FIGURE 3:**
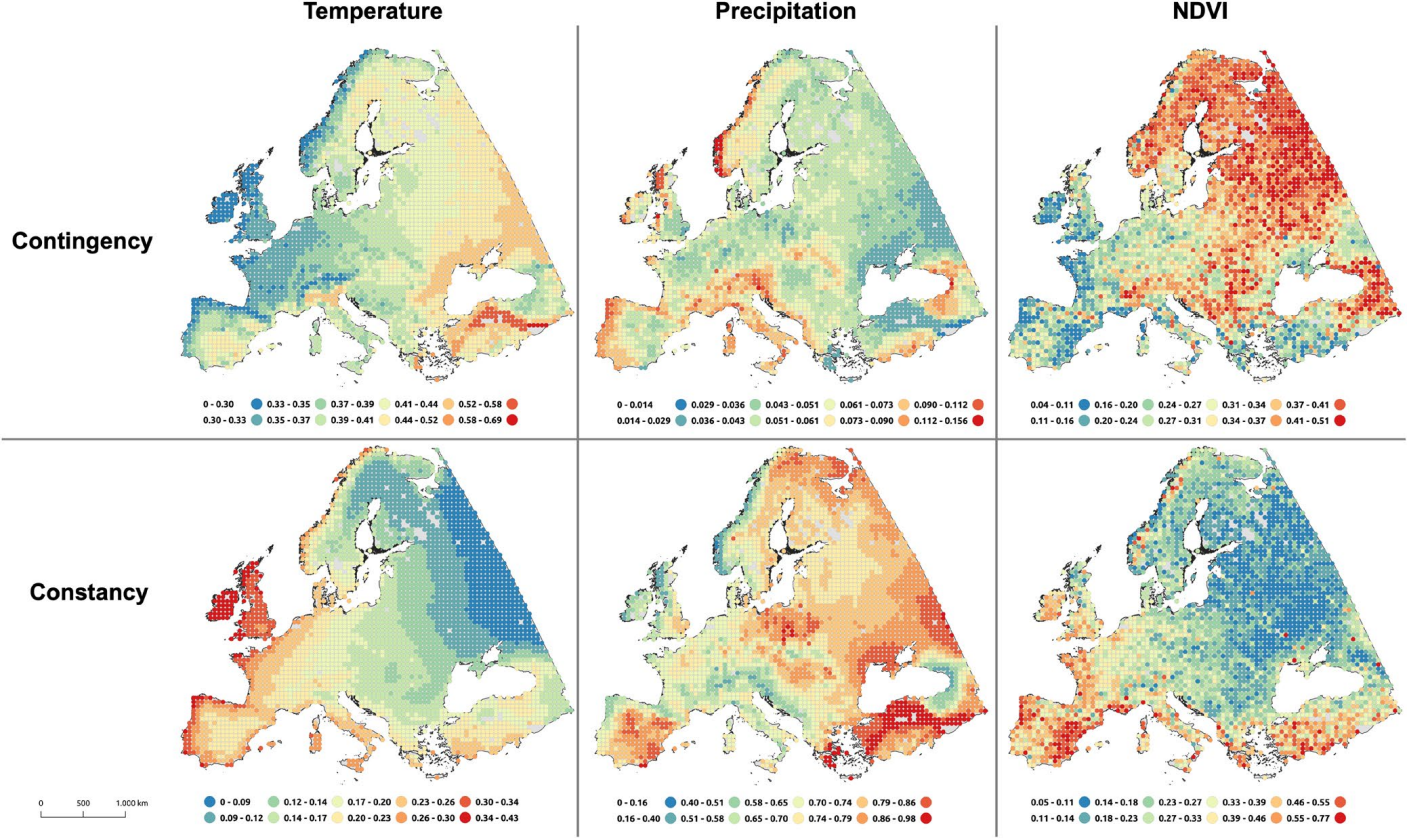
Contingency and constancy (possible range 0–1, with 0 representing no contingency/constancy and 1 representing complete contingency/constancy) of daily mean air temperature, precipitation and NDVI based on Colwell's (1974) metrics (see Figure 1), using a spatial grid resolution of 50 km across Europe, with point measurements represented by a single point per grid cell.

We mapped contingency and constancy values with respect to mean air temperature, total precipitation and NDVI for all grid points across Europe (Figure 3). With respect to mean temperature, we observed clear gradients from both west to east (Atlantic to continental) and from south to north. Analogously, contingency and constancy of NDVI also followed a gradient from west to east, with contingency increasing and constancy decreasing along this gradient. Additionally, higher values of contingency and lower values of constancy were found for alpine regions. There were no clear spatial gradients of contingency or constancy for precipitation, and values of contingency for precipitation were relatively low across Europe. From the spatial distribution of values for constancy of precipitation, arid areas in Europe could be clearly identified.

### 3.2 Parturition phenology

**FIGURE 4:**
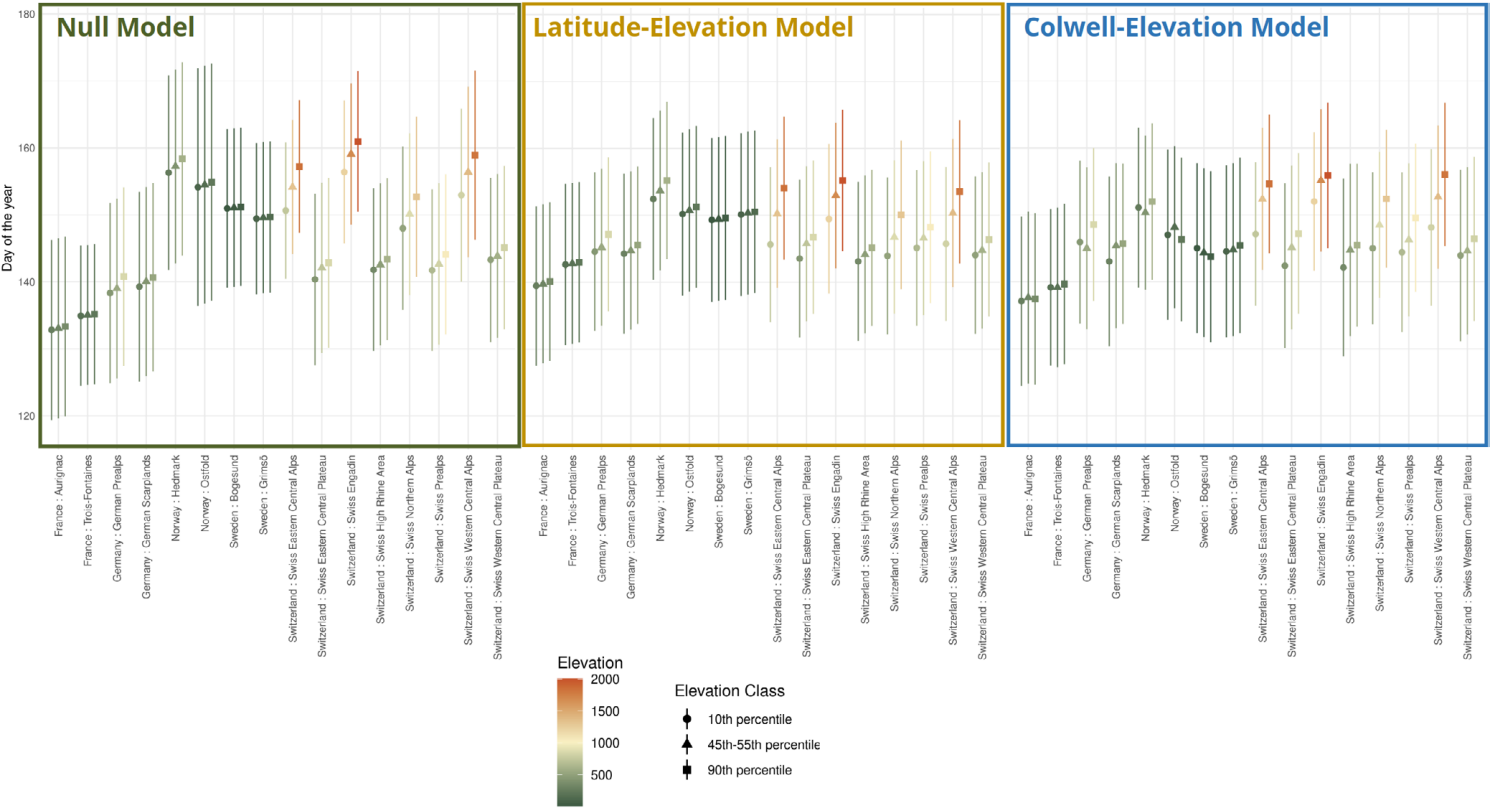
Predictions of the three models for the parturition phenology for each region, divided into three elevation classes corresponding to the 10th percentile (lowest), the 45th to 55th percentile (average) and the 90th percentile (highest) from the distribution of birth locations per region. Colours represent the absolute values for elevation at these quantiles.

Parturition dates across Europe followed two clear spatial gradients, with increasingly later births both from south to north, and from low to high elevations (Figure 2 and Table S1 in the Appendix). Births were earliest in Aurignac, in the south of France (median lower bound: Day of the Year (DoY) 129, median upper bound: DoY 134), and latest in Hedmark, Norway and Swiss Engadin (median lower bound: DoY 156–157; median upper bound: DoY 160–161). The highest synchrony in parturition timing was observed in the Swiss Eastern Central Alps (std = 8.4 days), while the lowest was in Ostfold, Norway (std = 14.7 days). The predictions for mean parturition date and synchrony (80% interval) per region are given in Figure S2 in the Appendix, and for corresponding elevation classes in Figure 4. The ‘Null Model’, which accounts for the effects of elevation and region on parturition date, can be regarded as a descriptive baseline of the data. The plots of the ‘Latitude‐Elevation Model’ and the ‘Colwell‐Elevation Model’ can be considered as fine‐scale adjustments to this baseline, accounting for the effects of either latitude or environmental predictability, respectively. The ‘Latitude‐Elevation Model’ and the ‘Colwell‐Elevation Model’ mirror the overall pattern of the ‘Null Model’; however, they did not accentuate the extremes of particularly early or late births to the same degree (see Aurignac or Hedmark and Ostfold). The results of the ‘Colwell‐only Model’ are given in Figure S3 in the Appendix for comparison. The ∆log‐likelihood compared to the ‘Null Model’ was −102.22 for ‘Latitude‐Elevation Model’ and −104.44 for the ‘Colwell‐Elevation Model’, indicating that model fit was somewhat improved by accounting for environmental predictability using metrics of constancy and contingency instead of latitude. However, while both the ‘Latitude‐Elevation Model’ (∆AIC: 73.78) and the ‘Colwell‐Elevation Model’ (∆AIC: 33.55) had higher log‐likelihoods compared to the ‘Null Model’, they were penalized for their greater complexity, resulting in higher AIC values, indicating that the ‘Null Model’ provided the most parsimonious description of the data.

The results of the sensitivity analysis using a transformation model with no assumptions of data distribution generated slightly wider 80% confidence intervals, indicating more variance than under the normality assumption. The mean values for parturition date and the spatial variation across the different regions, however, remained relatively unchanged, indicating that the results were robust to assumptions of normality (see Figure S4 in the Appendix).

#### 3.2.1 Predictions for variation in birth date across the roe deer distributional range

**FIGURE 5:**
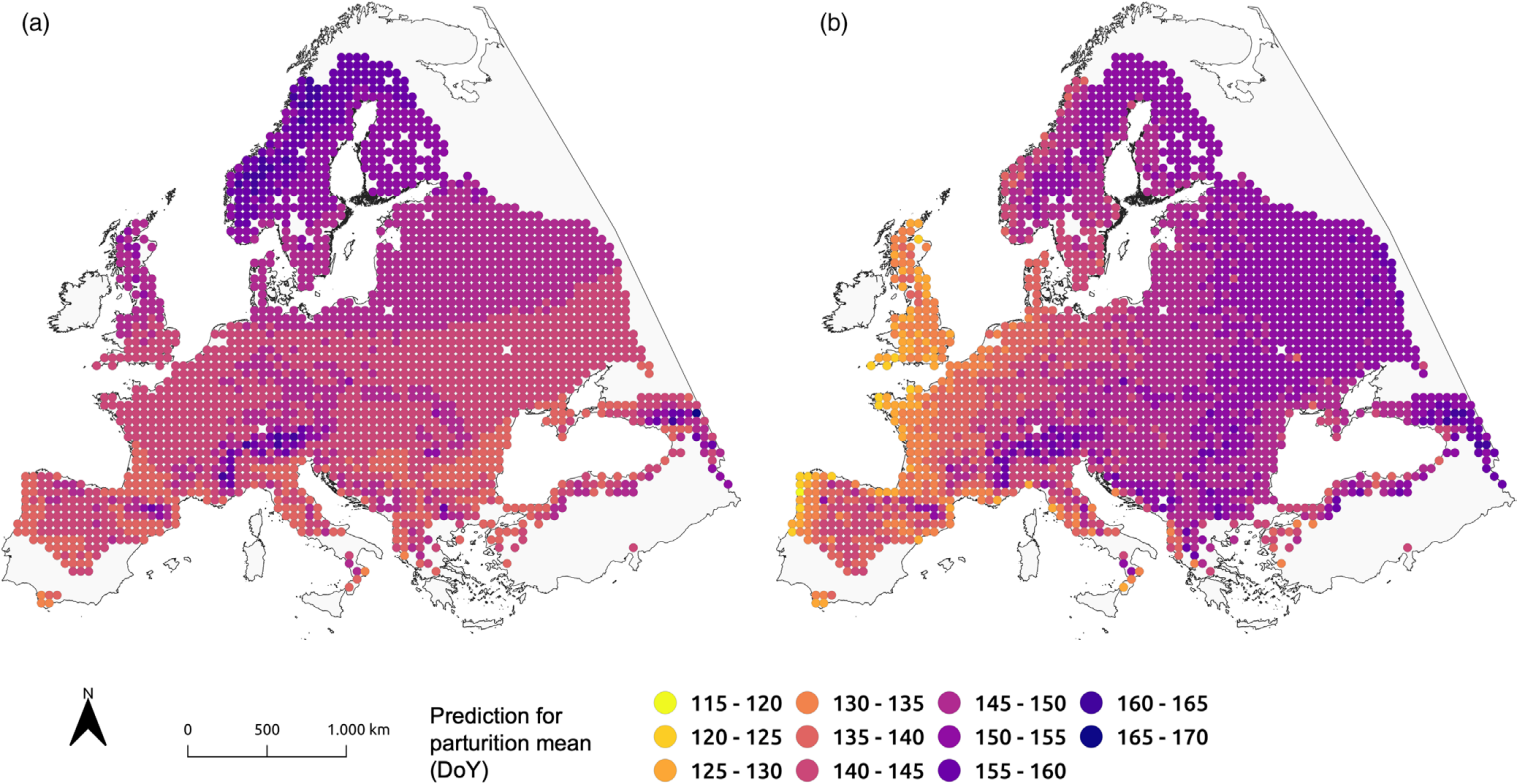
Predictions for mean parturition date (DoY) as described by the (a) ‘Latitude‐Elevation Model’ and the (b) ‘Colwell‐Elevation Model’ for each 50 km grid point within the roe deer distribution across Europe.

**FIGURE 6:**
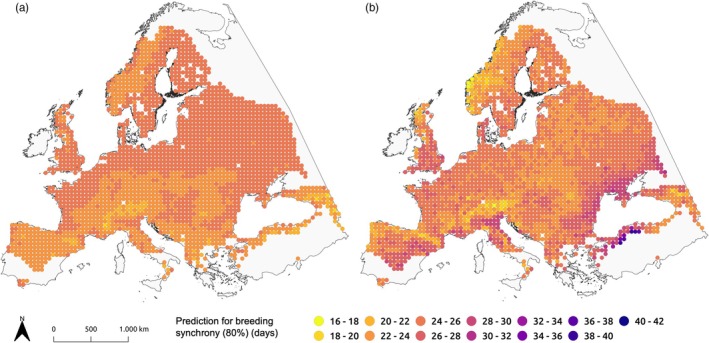
Predictions for parturition synchrony (80% interval, in days) based on the (a) ‘Latitude‐Elevation Model’ and the (b) ‘Colwell‐Elevation Model’ for each 50 km grid point within the roe deer distribution across Europe.

Next, we visualized the predicted values for mean parturition date and synchrony for all grid points within the distributional range of roe deer across Europe (Figures 5 and 6). For the predicted mean parturition timing (Figure 5), the three models generated different spatial patterns. With the ‘Latitude‐Elevation Model’, parturition timing was uniformly later at higher latitudes, with clear linear gradations (minimum predicted parturition date: DoY 133, mean: DoY 146, maximum: DoY 170, std.: 5.15 days). With the ‘Colwell‐Elevation Model’, parturition timing was increasingly later from west to east and was also slightly later from south to north (minimum: DoY 118, mean: DoY 146, maximum: DoY 163, std.: 6.98 days).

Based on the ‘Latitude‐Elevation Model’, the earliest predicted mean parturition date was DoY 133 in southern Spain, and the latest was DoY 170 in the Caucasus Mountains. In comparison, the earliest mean parturition date based on the ‘Colwell‐Elevation Model’ was predicted to be DoY 118 on the coast of Northwest Spain, while the latest was predicted to occur on DoY 163, also in the Caucasus Mountains. Generally, both models predicted that parturition timing would be later in mountainous regions such as the Alps, the Carpathian Mountains, the Caucasus or the Pyrenees.

With respect to parturition synchrony (Figure 6), the ‘Latitude‐Elevation Model’ predicted an approximately uniform pattern across Europe (minimum predicted parturition synchrony: 18 days, mean: 24 days, maximum: 25 days, std.: 0.73 days), but with greater parturition synchrony in mountainous regions. The most synchronized parturition was predicted to occur in the Caucasus Mountains and the Alps, while the most asynchronous was predicted to occur along the coastal areas of Europe with the lowest elevations, but there was no detectable latitudinal trend in synchrony. Based on the ‘Colwell‐Elevation Model’ (minimum predicted parturition synchrony: 17 days, mean: 25 days, maximum: 40 days, std.: 1.92 days), the most synchronized birth seasons were predicted to occur in the Alps and on the west coast of Norway, while the widest range of births within a season was expected to occur in Northern Türkiye.

#### 3.2.2 Validation of model predictions for parturition date and synchrony

Finally, we compared the predictive power of the above two models for describing the empirical distribution of parturition dates and synchrony across Europe using an independent data set from previously published data in the literature (Table 1). For mean parturition date, the ‘Latitude‐Elevation Model’ predicted the mean dates incorrectly by up to 13 days (England and Norway, Storfosna), with a mean absolute error (MAE) of 7.91 days. In comparison, the ‘Colwell‐Elevation Model’ predicted parturition to occur 1–8 days earlier than the reported mean/median across Europe, except for Germany (Baden‐Wuerttemberg), where it predicted parturition to occur 4 days later. The MAE of the predictions was 4.37 days. The differences between the models, particularly regarding the west‐to‐east gradient highlighted in Figure 5, were also evident here: whereas the ‘Latitude‐Elevation Model’ predicted the mean parturition date in England, Chedington, to be 24 May (DoY 144; 13 days later than reported), the ‘Colwell‐Elevation Model’ predicted 7 May (DoY 127; four days earlier than reported). Similarly, divergent predictions were obtained for Storfosna and Jøa in Norway. The predictions for parturition synchrony (80% interval) for the ‘Latitude‐Elevation Model’ differed by up to six days in comparison with observed parturition synchrony from the literature (MAE: 2.91 days). However, for four of the seven reported observations, this model yielded an estimate that was accurate within 2 days. The ‘Colwell‐Elevation Model’ yielded slightly poorer results (MAE: 3.69 days), with the highest divergence of nine days in Chedington Woods, in England. Again, however, for four of the seven reported observations, model predictions were accurate to within 3 days, with two observations perfectly predicted.

## 4 DISCUSSION

Intra‐specific variation in the timing of life‐history events across a species' distributional range and the potential underlying drivers is integral to understanding how rapid environmental change may impact populations in the wild. In this large‐scale study, we modelled the parturition phenology of roe deer in relation to elevation and the locally experienced environmental predictability, and then predicted birth date variation across its entire distributional range in Europe. We investigated whether metrics of weather and forage‐related contingency and constancy, together with elevation, yielded more robust predictions for roe deer parturition phenology than using the usual combination of latitude and elevation as proxies for environmental gradients. Our findings indicate, as expected for an income breeder, that roe deer align their mean parturition date with the long‐term predictability of environmental conditions and elevational gradients.

As expected, parturition was predicted to occur increasingly later from west to east across the roe deer's distributional range in Europe, consistent with gradients of more stable and longer windows of suitable weather conditions for plant growth in the west compared to the east. Parturition was also predicted to occur later at higher elevations, reflecting the delayed onset of spring and shorter growing seasons in mountainous regions. Specifically, the predictions generated by our approach for mean parturition date in pivotal and representative regions such as in the United Kingdom and for the Norwegian coastal areas, which are characterized by their constant and mild climate, were in good agreement with field observations reported in previous studies (summarized in Table 1). Indeed, the Atlantic meridional overturning circulation (AMOC), and its associated North Atlantic Current, generate these relatively mild and constant climates at higher western European latitudes (Figure 3), equally affecting the progress of spring across Europe (Menzel et al., 2005). This leads to higher constancy in temperature along the coastal regions of western Europe, a pattern reinforced by low elevation, resulting in earlier spring phenology (Ahas et al., 2002), which drives the earlier predicted parturition dates in these regions. Similar observations were made by Linnell and Andersen (1998), who found a pronounced difference in parturition dates of 14 days between the Norwegian coastal areas (study regions of Jøa and Storfosna) and the inland Hedmark population, despite an elevation difference of less than 400 m. Indeed, this substantial difference in birth timing is unlikely to be explained by elevation alone, as Peláez et al. (2020) suggested a delay in parturition timing of about 0.7 days per 100 m gain in altitude. Thus, when accounting for elevational gradients, environmental predictability appears to be a stronger predictor of parturition date than latitude. Similarly, with respect to elevation, in Switzerland, Peláez et al. (2020) found a stronger correlation between spring plant phenology and parturition timing than between parturition timing and elevation per se.

High parturition synchrony is often interpreted as an anti‐predator tactic (predator swamping; Estes, 1976). However, this is less likely in the case of a generalist predator, such as the red fox (*Vulpes vulpes*), the principal predator of roe deer fawns, which switches opportunistically between alternative prey (Kjellander & Nordström, 2003). Furthermore, as an income breeder, the roe deer is under strong selection pressure to match parturition to the peak of high‐forage availability in seasonal environments, which, accordingly, is expected to vary along a gradient of seasonality (Linnell & Andersen, 1998). Therefore, we hypothesized that parturition synchrony would be highest in regions with high levels of seasonality, expressed as environmental contingency. High elevations, for example, displayed a higher level of phenological contingency (see Figure 3), and this link was clearly evident in the predictions of both the ‘Latitude‐Elevation Model’ and the ‘Colwell‐Elevation Model’ (Figure 6), and also corresponded closely with previous findings (Peláez et al., 2020). In contrast, as expected, we found no clear pattern in parturition synchrony in relation to latitude. Under the aforementioned premise that contingency and elevation are the main drivers of parturition synchrony, this observation can be attributed to the fact that the level of seasonality in temperature, precipitation and NDVI does not strictly follow latitudinal clines across Europe (see Figure 3). This is consistent with previous findings in roe deer (Peláez et al., 2020). Instead, we suggest that parturition synchrony is more strongly influenced by local environmental predictability. For instance, on the islands of Storfosna and Jøa in Norway, lower parturition synchrony was observed than in Baden‐Wuerttemberg, Germany, roughly 15 degrees of latitude further south (Table 1). In particular, the markedly oceanic and low‐elevation regions in Norway display high constancy in temperature, as well as early onset of green‐up (Karlsen et al., 2009; Moen, 1999; Wielgolaski et al., 2011). However, observed parturition synchrony in the more inland study region of Hedmark, Norway, was also surprisingly low. In contrast, the model predictions for parturition synchrony for Jøa and Storfosna (Norwegian coastal areas) were better when accounting for both environmental predictability and elevation. Similarly, in red deer (*Cervus elaphus*), parturition was more synchronized at the phenologically less predictable study site in France compared to Norway (Loe et al., 2005).

These results concerning the rather inconsistent link between parturition synchrony and environmental predictability indicate that other driving factors should be considered. Habitats with a high diversity of natural land cover types (forest, meadows, agriculture, shrubs, or moorland) or a high plant species diversity may extend the window of optimal forage conditions and, as a result, prolong the birth season (Brunot et al., 2025). These small‐scale heterogeneities in landscape composition, however, are likely not captured by the spatial resolution of most satellite sensors used to quantify the NDVI (Berra & Gaulton, 2021; Justice et al., 1985; White et al., 2014). Similarly, while the influence of elevation should theoretically also be reflected in Colwell's metrics, the coarse spatial resolution of the meteorological and phenological data likely limits their explanatory power. Hence, elevation remains a key predictor of birth phenology, particularly in steep terrain, where sharp climatic and phenological gradients are poorly reflected by the spatial resolution of the metrics (see  Figure S3 in the Appendix for model predictions from the ‘Colwell‐only Model’). This bias may also be amplified by the disproportionate sample size and, hence, influence of the Swiss data set, which spans a broad range of elevations. On the other hand, in mountainous areas, topographically diverse habitats could provide roe deer with the opportunity to track plant phenology of a variety of plant species with their respective phenologies along altitudinal gradients or variable snowmelt patterns, providing a longer period of optimal forage conditions and the avoidance of snow cover (e.g. in red deer; Albon & Langvatn, 1992; Mysterud et al., 2001; Loe et al., 2005). For example, the Scandinavian Caledonian mountain range rises abruptly on the oceanic‐influenced western Norwegian coast (Wielgolaski et al., 2011), creating diverse habitats at small spatial scales. Telemetry‐based studies have demonstrated partial migration in red deer (Rivrud et al., 2016) and in roe deer in mountainous regions such as Norway (Mysterud, 1999) or the Alps (Peters et al., 2017; Ramanzin et al., 2007). Building on this rationale, in addition to the temporal dimension of environmental predictability, the spatiotemporal scale and degree of landscape heterogeneity may also determine the behaviour and life‐history responses of large herbivores (Riotte‐Lambert & Matthiopoulos, 2020). For example, in heterogeneous habitats, animals can take advantage of the lower spatial synchrony in spring green‐up by opportunistically migrating (Cagnacci et al., 2011; Peters et al., 2017) or adopting a multi‐range tactic (Couriot et al., 2018) to extend the window of high‐quality forage.

Although our work is based on an unprecedentedly large sample of detailed data on parturition phenology in the wild, there are some shortcomings that should be noted. Firstly, despite the data set that was available for our analysis, small sample sizes for certain regions, heterogeneity in the accuracy of age estimation across sites and personnel, or variable and opportunistic sampling efforts likely all hamper accurate estimations of parturition synchrony (Kauffert et al., 2023; Peláez et al., 2020). Additionally, our data set included time series of different lengths and timing, which may also generate confounding effects (see Table S1 in the Appendix). For example, changes in habitat quality or composition over time in long‐term monitoring studies were not accounted for in our approach, but might lead to shifts in parturition timing (Gill, 1994), although the timing of birth in roe deer has been shown to vary little at the within‐individual level (Plard et al., 2013). Similarly, alterations in sex and age structures have been hypothesized to affect parturition synchrony in other large herbivores (Loe et al., 2005; Mysterud et al., 2002), but this is unlikely in the roe deer which has a single oestrous per year. The age and phenotypic quality composition of females within a population, and hence the management, might also influence breeding phenology (Langvatn et al., 2004; Loe et al., 2005) as older and high‐quality females tend to give birth earlier than their younger (2‐year‐old) and poorer quality counterparts (Plard, Gaillard, Coulson, Hewison, Delorme, Warnant, Nilsen, & Bonenfant, 2014). Finally, potential shifts in parturition timing in response to the earlier onset of spring (see Hagen et al., 2021; Rehnus et al., 2020) might inflate synchrony estimations. Still, a consistent and marked phenological shift in parturition timing in roe deer has not yet been demonstrated (Plard, Gaillard, Coulson, Hewison, Delorme, Warnant, & Bonenfant, 2014). To address these points to some extent, we incorporated a random effect, controlling for the interaction of region and year, and used interval‐censored parturition dates. Finally, from a technical point of view, our vegetation‐related remote sensing index (NDVI) is sensitive to cloud cover. Frequent, seasonal or persistent cloud coverage may have biased the estimates of contingency and constancy. Although we used robust smoothing and filtering techniques to address this issue, this limitation is particularly common across mountainous regions, further highlighting the need to explicitly consider elevation. Here, new radar sensors might track the vegetation flush better and are not affected by cloud cover (Bae et al., 2022).

Overall, it is evident that the level of parturition synchrony within populations of roe deer cannot be attributed to a single factor, and other components or confounding effects might also play important roles. Still, our study demonstrates that linking roe deer parturition phenology to environmental predictability and elevation yields more robust results compared to using the combination of latitude and elevation alone. Particularly, latitude is only a proxy for environmental gradients that obviously do not fully capture the underlying environmental mechanisms. Instead, directly relating parturition phenology to environmental data provides a clearer understanding of these underlying macrophenological processes. Nevertheless, it is essential to gather observations from the eastern and southeastern areas of the distributional range of roe deer to validate our models further across the species' range. These areas accentuate the extremes of environmental predictability within the roe deer's range and should be prioritized alongside ongoing systematic observations in established study regions to ensure long‐term and consistent time series.

## 5 CONCLUSION

Our analysis provides the first attempt to model and predict roe deer parturition phenology in relation to locally experienced environmental predictability and elevation. The phenology of parturition markedly influences female reproductive performance, making it crucial to understand these interactions for assessing trophic mismatches in response to changing climates (Helm et al., 2013; Visser & Both, 2005). Our results provide an important starting point to comprehend the macrophenological processes underlying variation in the timing of life‐history events across space. Further, they highlight the importance of accounting for this spatial variation when calculating region‐specific adaptations (Bailey et al., 2022). Given the heterogeneous nature of climate change exposure and the speed of plant phenological shifts across Europe (Ahas et al., 2002), region‐specific predictions will be of utmost importance (Bailey et al., 2022), particularly for species that inhabit wide geographical ranges. Sensitivity to environmental changes or novel climates might vary depending, for example, on factors such as the environmental predictability experienced (Bailey et al., 2022; Bitter et al., 2021; Leung et al., 2020), or triggering responses like opportunistic migration (Cagnacci et al., 2011). In sum, by elucidating the intricate relationship between roe deer parturition phenology and the combination of environmental predictability and elevation, our study enhances the understanding of ungulate adaptation and underscores the importance of region‐specific approaches to addressing the challenges posed by climate change.

## AUTHOR CONTRIBUTIONS

Johanna Kauffert, A. J. Mark Hewison, Benedikt Gehr, Torsten Hothorn, Sophie Baur, Jörg Müller, Wibke Peters and Annette Menzel conceptualized the work. Johanna Kauffert, A. J. Mark Hewison, Benedikt Gehr, Sophie Baur, Jean‐Michel Gaillard, Petter Kjellander, Andreas König, Maryline Pellerin, Manuela Panzacchi, Wibke Peters and Annette Menzel collected and curated the data. Johanna Kauffert, A. J. Mark Hewison, Benedikt Gehr, Torsten Hothorn, Jörg Müller, Balint Tamasi, Wibke Peters and Annette Menzel designed the methodology, and Johanna Kauffert and Torsten Hothorn performed the formal analysis with support from Balint Tamasi. Johanna Kauffert, Torsten Hothorn and Balint Tamasi wrote the software. Johanna Kauffert and Torsten Hothorn designed the visuals. Annette Menzel, Andreas König and Wibke Peters acquired funding. Annette Menzel led the project administration. Annette Menzel supervised the work with support from Torsten Hothorn, A. J. Mark Hewison and Wibke Peters. Johanna Kauffert led the writing of the manuscript with support from A. J. Mark Hewison, Jean‐Michel Gaillard and Annette Menzel. All authors contributed critically to the drafts and gave final approval for publication.

## CONFLICT OF INTEREST STATEMENT

The authors declare no competing interests.

## FUNDING INFORMATION

Johanna Kauffert, Sophie Baur, Annette Menzel, Andreas König and Wibke Peters were funded by the Bavarian Ministry of Food, Agriculture, and Forestry, grant number: A19/17. Torsten Hothorn received funding from the Swiss National Science Foundation SNF 200021_219384. Jean‐Michel Gaillard, A. J. Mark Hewison, and Maryline Pellerin were funded by the ‘DivinT’ Agence Nationale de la Recherche grant ANR‐22‐CE02‐0020‐03.

## Supporting information


**Figure S1.** Geographic range of roe deer in Europe (Lovari et al., 2016) displayed with the biogeographical regions (European Environment Agency, 2016).
**Table S1.** Overview of the study regions used for the analysis and the validation. The mean parturition date, the synchrony, the number of observations and the years of observation. The median contingency (M) and constancy (C) for temperature, precipitation and NDVI are provided as well as the median elevation of the study region.
**Figure S2.** Comparison of model predictions of the three models based on the full data set, with the underlying full data set represented as violin plots per region.
**Figure S3.** Predictions of the ‘Colwell‐only Model’ for the parturition phenology for each region, divided into three elevation classes corresponding to the 10th percentile (lowest), the 45th to 55th percentile (average) and the 90th percentile (highest) from the distribution of birth locations per region. Colours represent the absolute values for elevation at these quantiles. Compared to the ‘Null Model’, the model had a ∆log‐likelihood of −37.43 and a ∆AIC of 243.87.
**Figure S4.** Comparison of the predictions of the three models between the GAMLSS under the normality assumption and the non‐parametric transformation model (shift‐scale mixed effect model). The regions are divided into three elevation classes corresponding to the elevation of the 10th percentile of the lowest, 45th to 55th percentile and the 90th percentile of the highest parturition locations reported per region. Colours represent the model framework. Colours represent the model framework.

**Data S1.** Quantile Regression for Switzerland.

## Data Availability

The elevation data were extracted from ALOS World 3D‐30m (AW3D30) (Takaku et al., 2020). NDVI data were extracted from MODIS Terra Surface Reflectance 8‐Day Global 250 m, MOD09Q1. MODIS and AW3D30 data were accessed via Python's Google Earth Engine API (Gorelick et al., 2017; https://developers.google.com/earth‐engine/datasets/catalog/JAXA_ALOS_AW3D30_V4_1; https://developers.google.com/earth‐engine/datasets/catalog/MODIS_061_MOD09Q1). Total daily precipitation (mm) and mean temperature (°C) data were extracted from the E‐OBS Gridded Dataset (v26.0) (Cornes et al., 2018; https://surfobs.climate.copernicus.eu/dataaccess/access_eobs.php#datafiles). The roe deer fawn data are available from the corresponding author upon reasonable request. Code is available from Figshare https://figshare.com/s/9be6f9a7f12c8f546ad0 (Kauffert, Hewison, et al., 2025).
