# Supplementary material for: Synching with seasonality: Predicting roe deer parturition phenology across its distributional range
Source: J Anim Ecol. 2025 Oct 3;94(12):2542–57. doi: 10.1111/1365-2656.70148 (PMC12673248; doi:10.1111/1365-2656.70148)
Supplement: Supplementary file 1 — Figure S1. Geographic range of roe deer in Europe (Lovari et al., 2016) displayed with the biogeographical regions (European Environment Agency, 2016). Table S1. Overview of the study regions used for the analysis and the validation. The mean parturition date, the synchrony, the number of observations and the years of observation. The median contingency (M) and constancy (C) for temperature, precipitation and NDVI are provided as well as the median elevation of the study region. Figure S2. Comparison of model predictions of the three models based on the full data set, with the underlying full data set represented as violin plots per region. Figure S3. Predictions of the ‘Colwell‐only Model’ for the parturition phenology for each region, divided into three elevation classes corresponding to the 10th percentile (lowest), the 45th to 55th percentile (average) and the 90th percentile (highest) from the distribution of birth locations per region. Colours represent the absolute values for elevation at these quantiles. Compared to the ‘Null Model’, the model had a ∆log‐likelihood of −37.43 and a ∆AIC of 243.87. Figure S4. Comparison of the predictions of the three models between the GAMLSS under the normality assumption and the non‐parametric transformation model (shift‐scale mixed effect model). The regions are divided into three elevation classes corresponding to the elevation of the 10th percentile of the lowest, 45th to 55th percentile and the 90th percentile of the highest parturition locations reported per region. Colours represent the model framework. Colours represent the model framework. [file JANE-94-2542-s002.pdf]

Appendix

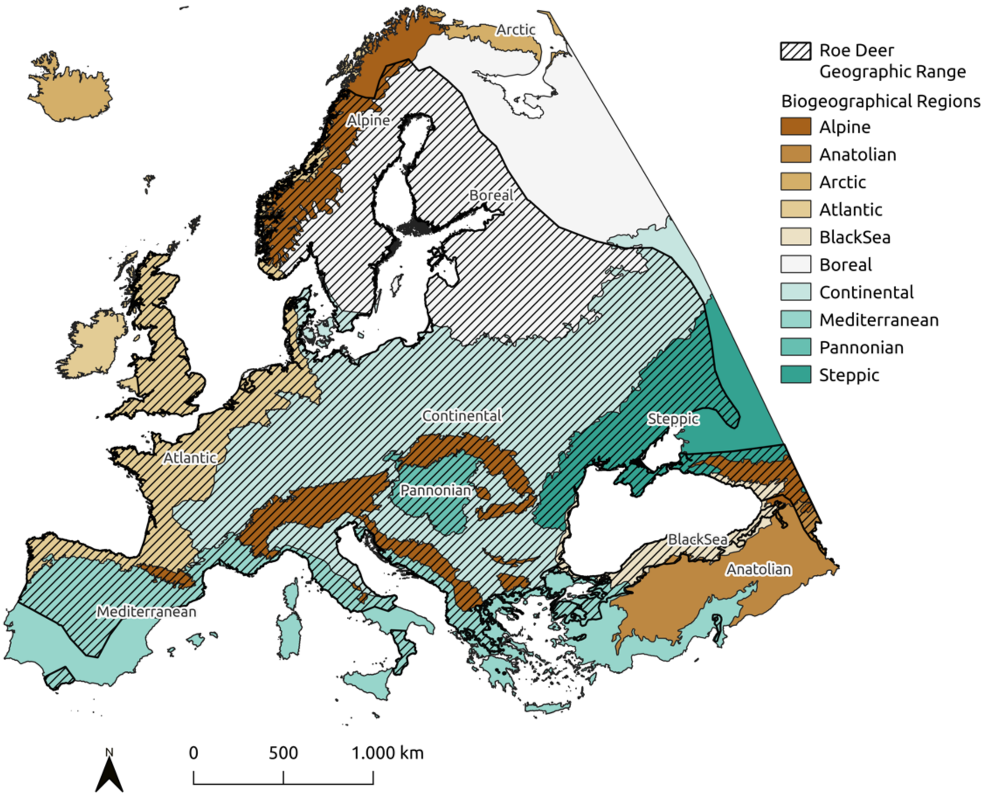

Figure S1. Geographic range of roe deer in Europe (Lovari et al., 2016) displayed with the biogeographical regions (European Environment Agency, 2016).

Table S1. Overview of the study regions used for the analysis and the validation. The mean parturition date, the synchrony, the number of observations and the years of observation. The median contingency (M) and constancy (C) for temperature, precipitation and NDVI are provided as well as the median elevation of the study region.

Study Region	Mean	Synchrony	n	Years	C* Temperature	M# Temperature	C Precipitation	M Precipitation	C NDVI	M NDVI	Elevation	Included in
Norway : Hedmark	157	25	70	1995–1998	0.14	0.37	0.62	0.05	0.14	0.37	408	Analysis
Norway : Ostfold	154	32	124	2001–2005	0.16	0.36	0.58	0.05	0.10	0.34	155.5	Analysis
Sweden : Grimsö	149	25	275	1997–2020	0.17	0.37	0.67	0.04	0.19	0.28	110	Analysis
Sweden : Bogesund	151	27	115	2013–2016	0.17	0.38	0.73	0.04	0.17	0.29	22	Analysis
Germany : German Scarplands	140	29	416	2020–2021	0.18	0.34	0.66	0.04	0.22	0.22	413	Analysis
France : Trois-Fontaines	135	22	809	1985–2011	0.22	0.32	0.61	0.04	0.28	0.32	188	Analysis
Germany : German Prealps	140	25	526	2020–2021	0.18	0.34	0.57	0.06	0.22	0.25	543.5	Analysis
Switzerland : Swiss High Rhine Area	143	25	98	1971–2020	0.19	0.35	0.49	0.06	0.27	0.23	469	Analysis
Switzerland : Swiss Eastern Central Plateau	142	27	2596	1971–2020	0.20	0.34	0.49	0.07	0.27	0.22	721	Analysis
Switzerland : Swiss Prealps	143	25	837	1971–2020	0.20	0.32	0.49	0.06	0.24	0.28	807	Analysis
Switzerland : Swiss Western Central Plateau	144	26	1150	1971–2020	0.19	0.35	0.52	0.06	0.27	0.23	591.5	Analysis
Switzerland : Swiss Northern Alps	150	26	1248	1971–2020	0.21	0.32	0.44	0.08	0.16	0.33	865	Analysis

Switzerland : Swiss Eastern Central Alps	154	21	5958	1972–2020	0.21	0.32	0.46	0.07	0.16	0.37	1331	Analysis
Switzerland : Swiss Engadin	159	22	2209	1973–2020	0.21	0.32	0.62	0.05	0.13	0.38	1690	Analysis
Switzerland : Swiss Western Central Alps	156	25	434	1998–2020	0.21	0.32	0.59	0.05	0.14	0.38	1387	Analysis
France : Aurignac	133	29	348	2004–2021	0.25	0.34	0.64	0.05	0.30	0.17	308	Analysis
Norway : Jøa	142	28	45	1992-1993	0.24	0.32	0.43	0.07	0.09	0.40	7	Validation
Norway : Storfosna	142	26	296	1991-1994	0.27	0.31	0.53	0.05	0.12	0.34	310	Validation
Sweden : Ekenaes	153	25	233	1986 - 1999	0.18	0.38	0.73	0.04	0.14	0.31	56	Validation
England : Chedington	131	18	42	1968 - 1972	0.33	0.30	0.62	0.04	0.42	0.09	109	Validation
Germany : Baden-Wuerttemberg	141	22	16130	1973-2019	0.19	0.32	0.60	0.04	0.20	0.33	493	Validation
Poland : Czempin	153		129	1976 - 1980	0.17	0.36	0.75	0.04	0.17	0.20	78	Validation
Italy : South Tyrol - Hahnebaum	164	21	113	1983-1992	0.20	0.31	0.55	0.07	0.13	0.34	1763	Validation
Italy : Apenines	150	17	117	1997 - 2003	0.19	0.37	0.56	0.07	0.20	0.34	687	Validation
Spain : Madrid	139				0.21	0.40	0.80	0.05	0.49	0.11	571	Validation

*C = constancy #M = contingency

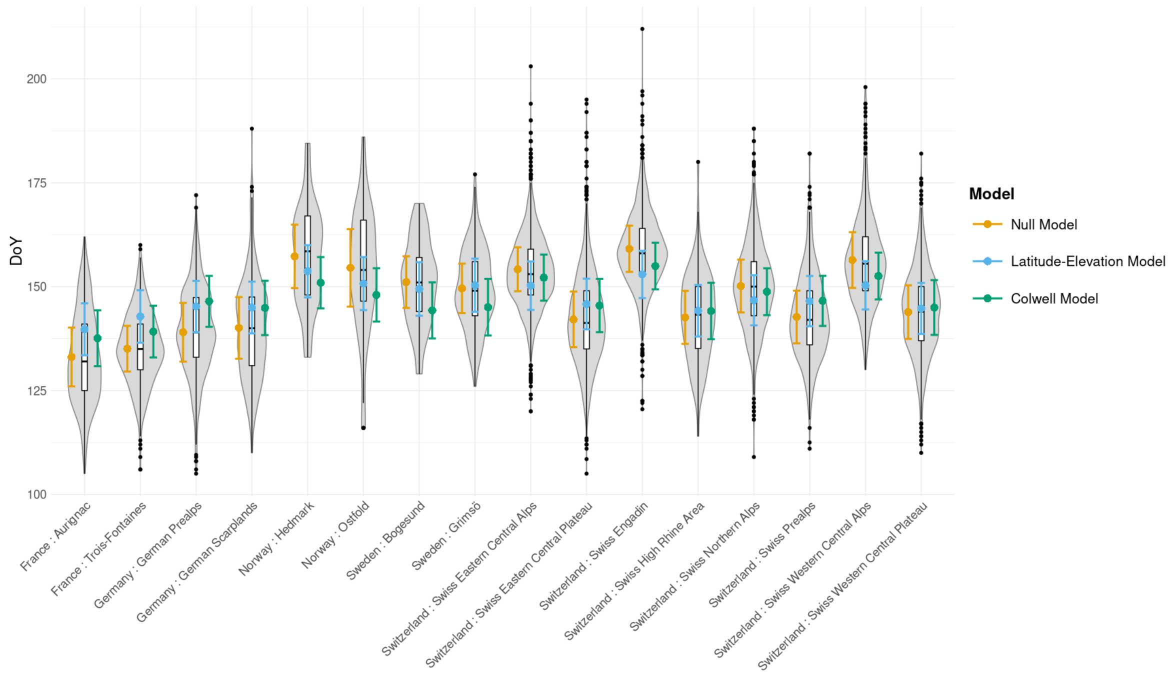

Figure S2. Comparison of model predictions of the three models based on the full data set, with the underlying full data set represented as violin plots per region.

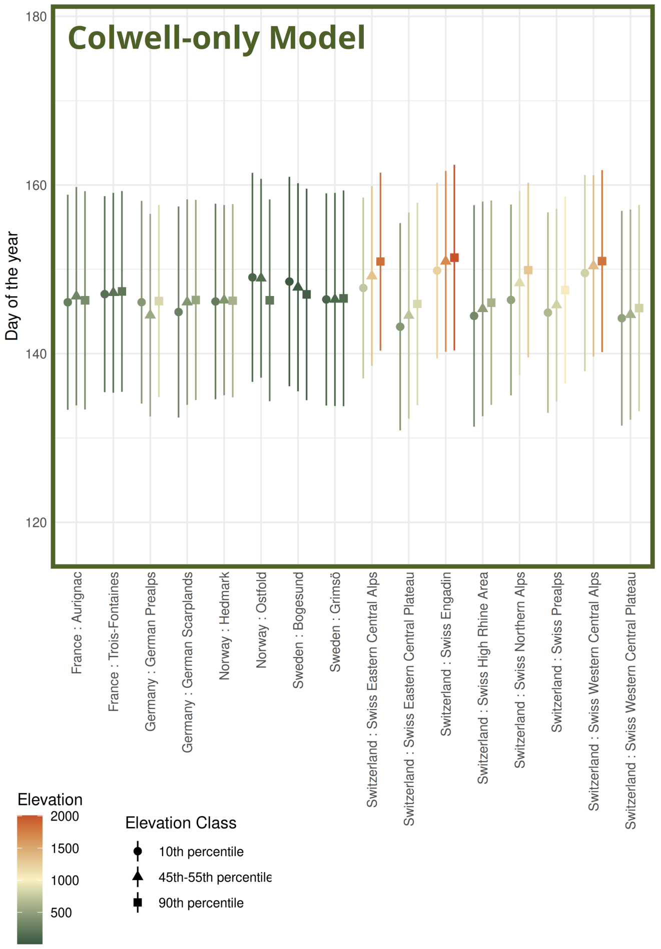

Figure S3. Predictions of the “Colwell-only Model” for the parturition phenology for each region, divided into three elevation classes corresponding to the 10th percentile (lowest), the 45th to 55th percentile (average), and the 90th percentile (highest) from the distribution of birth locations per region. Colors represent the absolute values for elevation at these quantiles. Compared to the “Null Model”, the model had a ∆log-likelihood of –37.43 and a ∆AIC of 243.87.

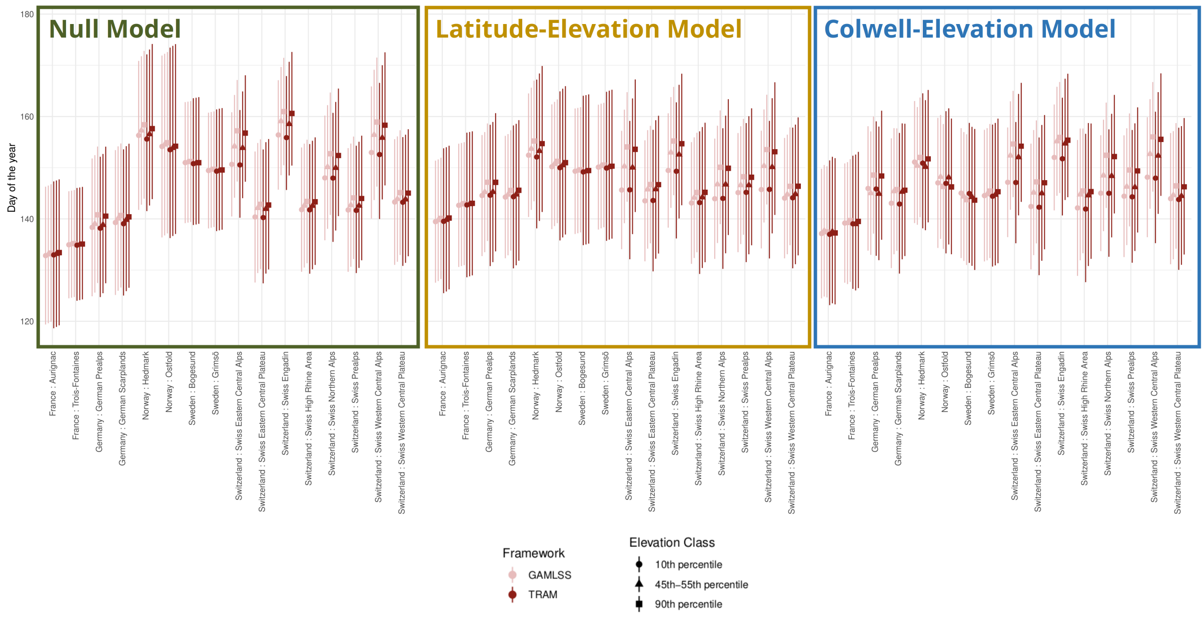

Figure S4. Comparison of the predictions of the three models between the GAMLSS under the normality assumption and the non-parametric transformation model (Shift-scale mixed effect model). The regions are divided into three elevation classes corresponding to the elevation of the 10th percentile of the lowest, 45th to 55th percentile, and the 90th percentile of the highest parturition locations reported per region. Colors represent the model framework. Colours represent the model framework.

References

European Environment Agency (2016). Biogeographical regions in Europe. https://www.eea.europa.eu/data-and-maps/figures/biogeographical-regions-in-europe-2.

Lovari, S., Herrero, J., Masseti, M., Ambarli, H., Lorenzini, R., and Giannatos, G. (2016). Capreolus capreolus. The IUCN Red List of Threatened Species 2016. e.T42395A22161386. https://dx.doi.org/10.2305/IUCN.UK.2016-1.RLTS.T42395A22161386.en.
