# Supplementary material for: Synching with seasonality: Predicting roe deer parturition phenology across its distributional range
Source: J Anim Ecol. 2025 Oct 3;94(12):2542–57. doi: 10.1111/1365-2656.70148 (PMC12673248; doi:10.1111/1365-2656.70148)

Quantile Regression for Switzerland

```
Switzerland : Swiss Western Central Plateau

   quantile   Intercept     Slope       p-value
0       0.1  441.000009 -0.151515  2.090356e-06
1       0.5  454.239079 -0.152174  2.984483e-08
2       0.9   82.250009  0.041667  3.896482e-01
```

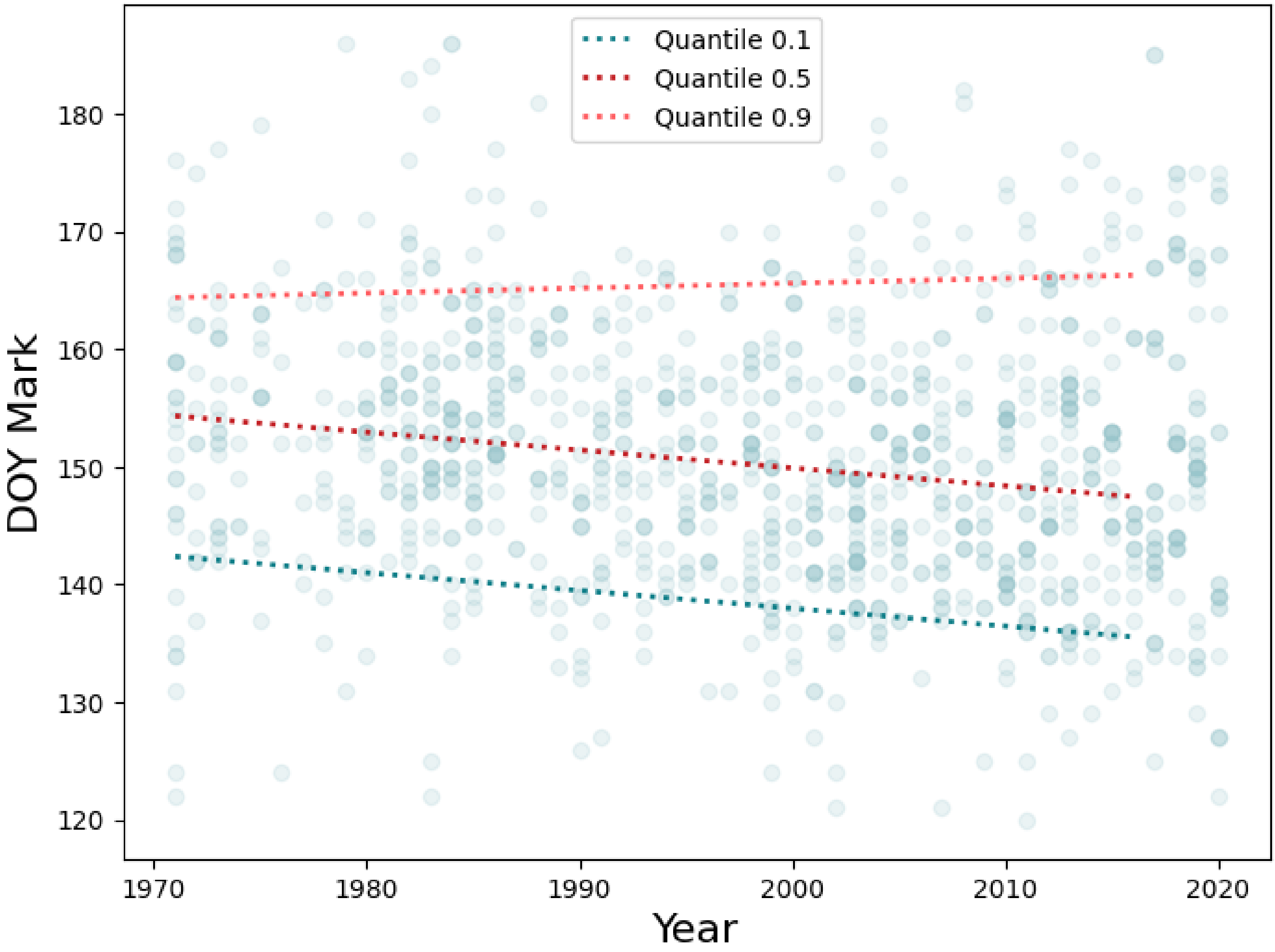


```
Switzerland : Swiss Eastern Central Plateau
```

	quantile	Intercept	Slope	p-value
0	0.1	289.384659	-0.076923	2.226454e-04
1	0.5	330.181760	-0.090909	2.146948e-07
2	0.9	97.266648	0.033333	2.223864e-01

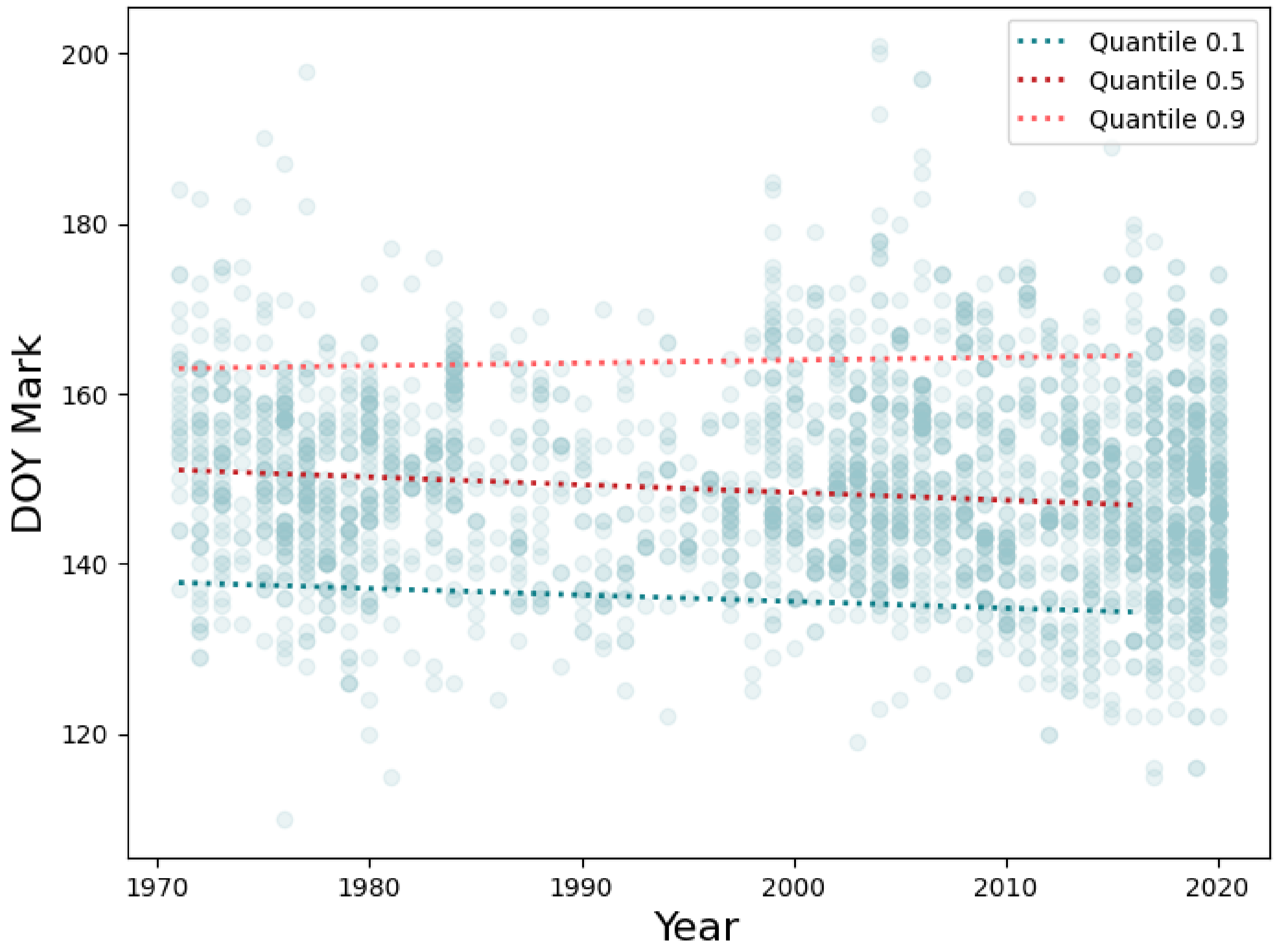

Switzerland : Swiss Jura and Randen

	quantile	Intercept	Slope	p-value
0	0.1	635.250028	-0.250000	1.213597e-12
1	0.5	488.489450	-0.170213	1.111282e-06
2	0.9	910.375006	-0.375000	1.867421e-16

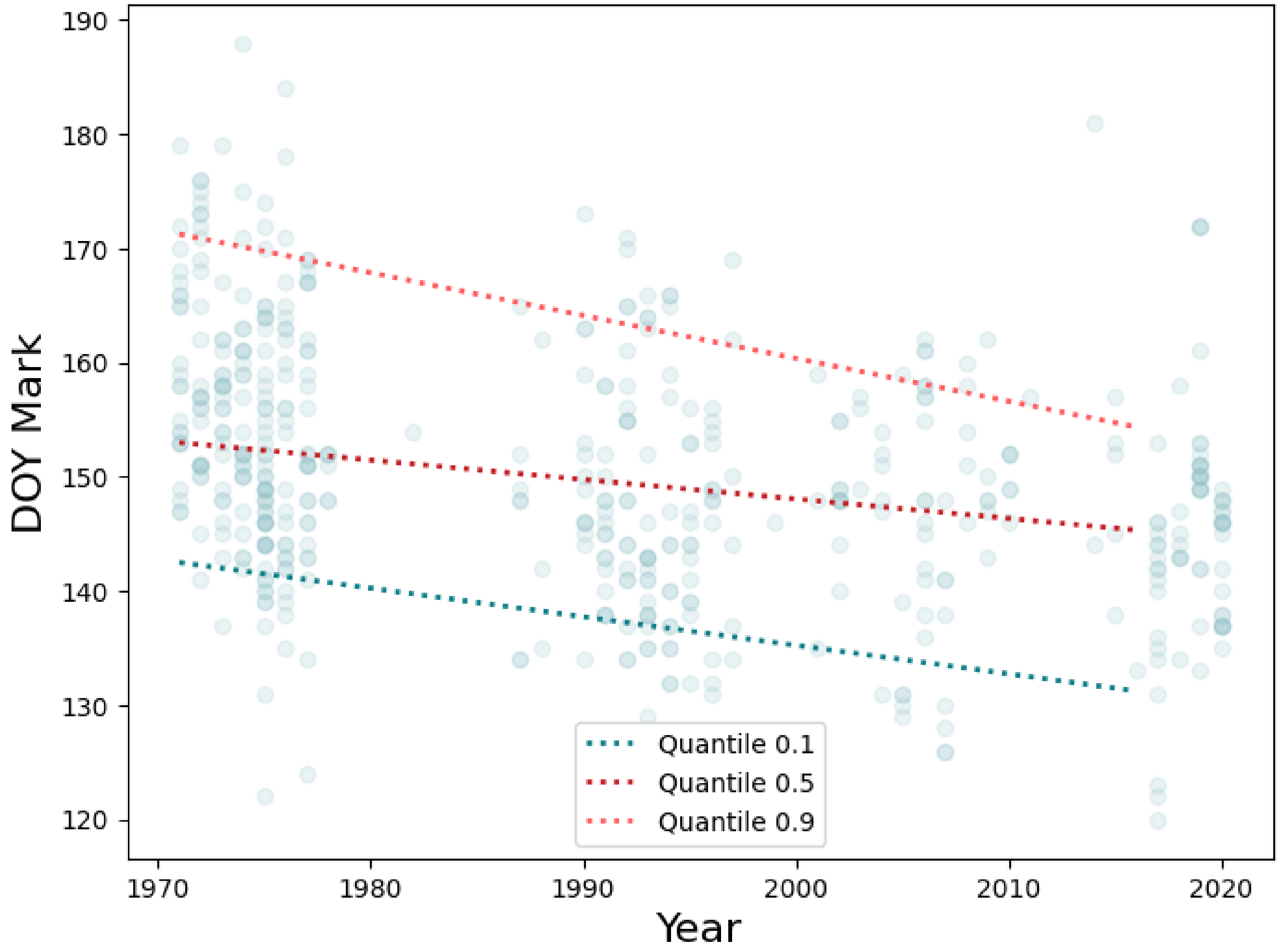

Switzerland : Swiss Northern Alps

	quantile	Intercept	Slope	p-value
0	0.1	237.000519	-0.047619	0.207938
1	0.5	365.578976	-0.105263	0.000019
2	0.9	110.794116	0.029412	0.478408

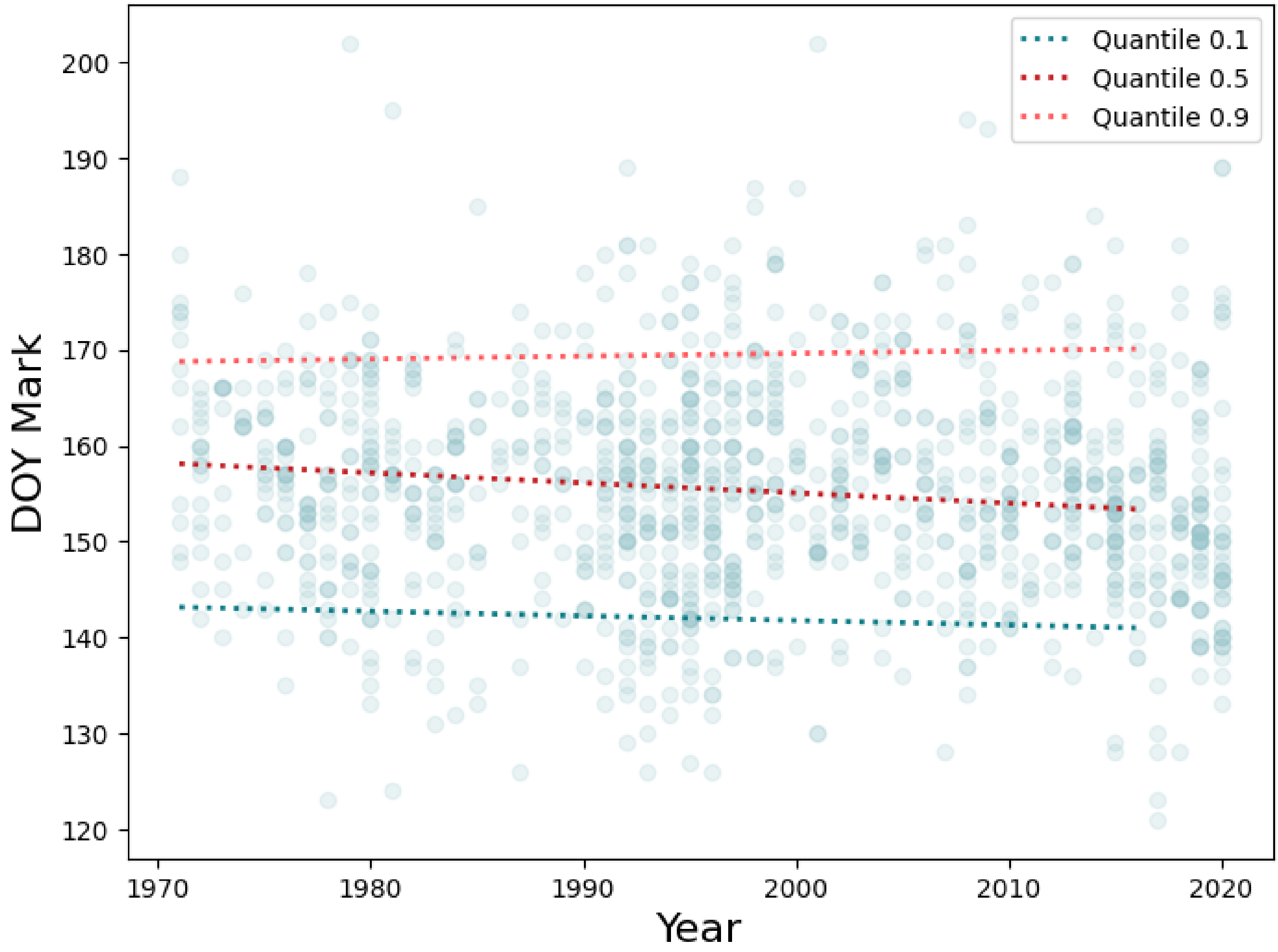

Switzerland : Swiss Prealps

	quantile	Intercept	Slope	p-value
0	0.1	279.857142	-0.071429	0.025948
1	0.5	378.884726	-0.115385	0.000031
2	0.9	363.718574	-0.100464	0.071217

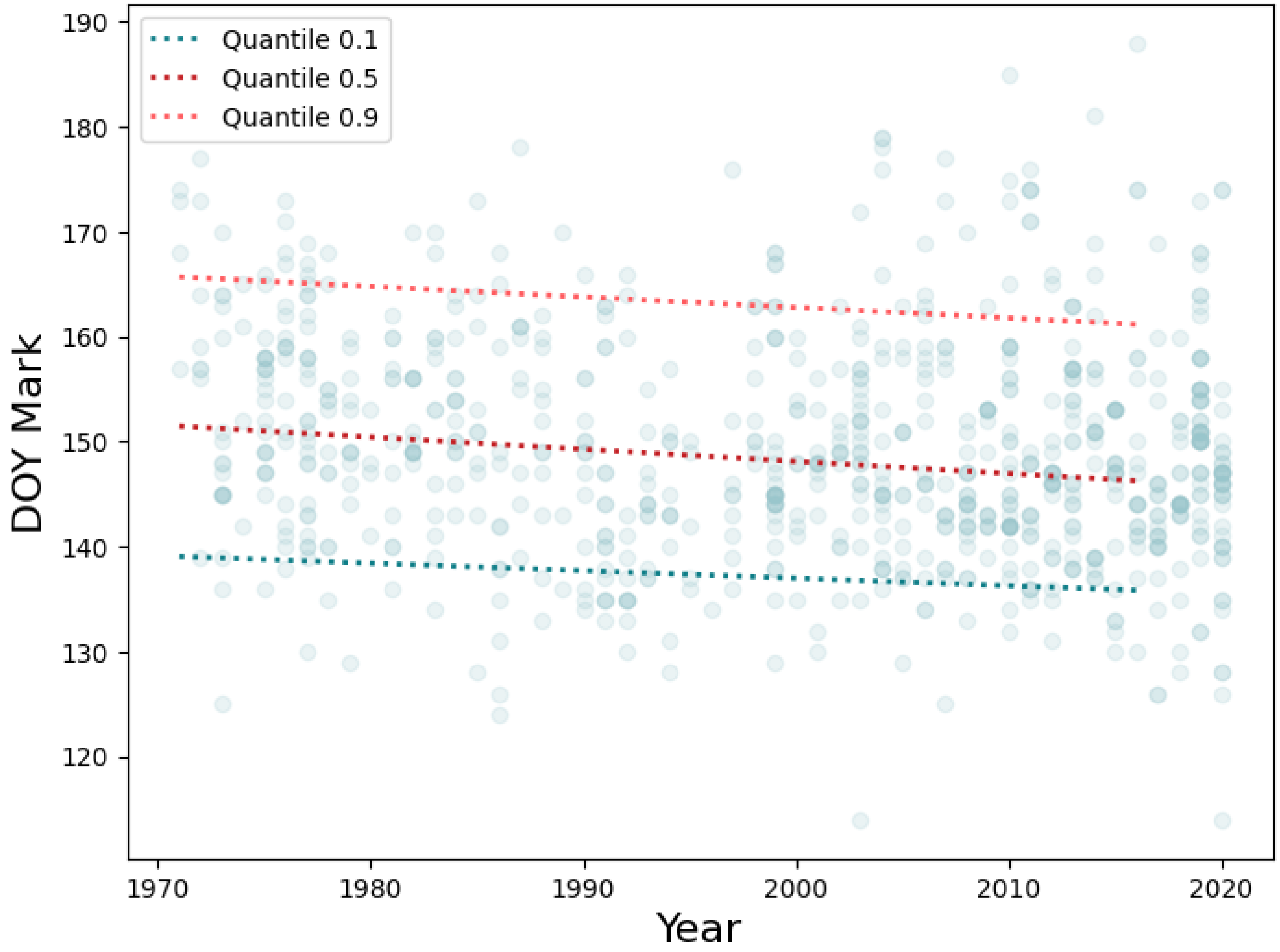

Switzerland : Swiss High Rhine Area

	quantile	Intercept	Slope	p-value
0	0.1	0.000036	0.070850	0.651144
1	0.5	645.750186	-0.250000	0.051478
2	0.9	738.451717	-0.290323	0.113040

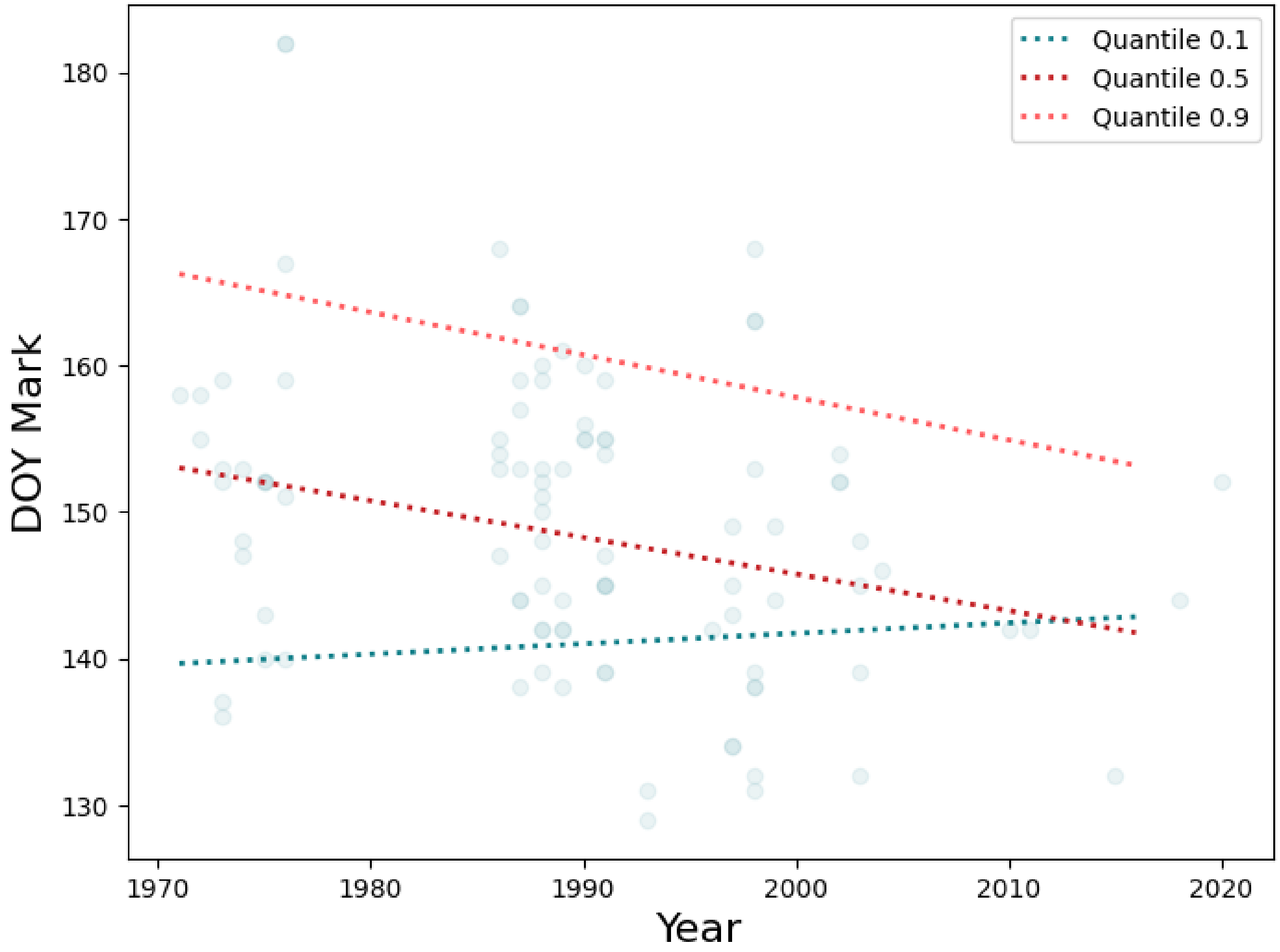

Switzerland : Swiss Eastern Central Alps

	quantile	Intercept	Slope	p-value
0	0.1	370.888872	-0.111111	3.720929e-10
1	0.5	381.777824	-0.111111	6.171269e-17
2	0.9	250.760072	-0.040000	9.755633e-02

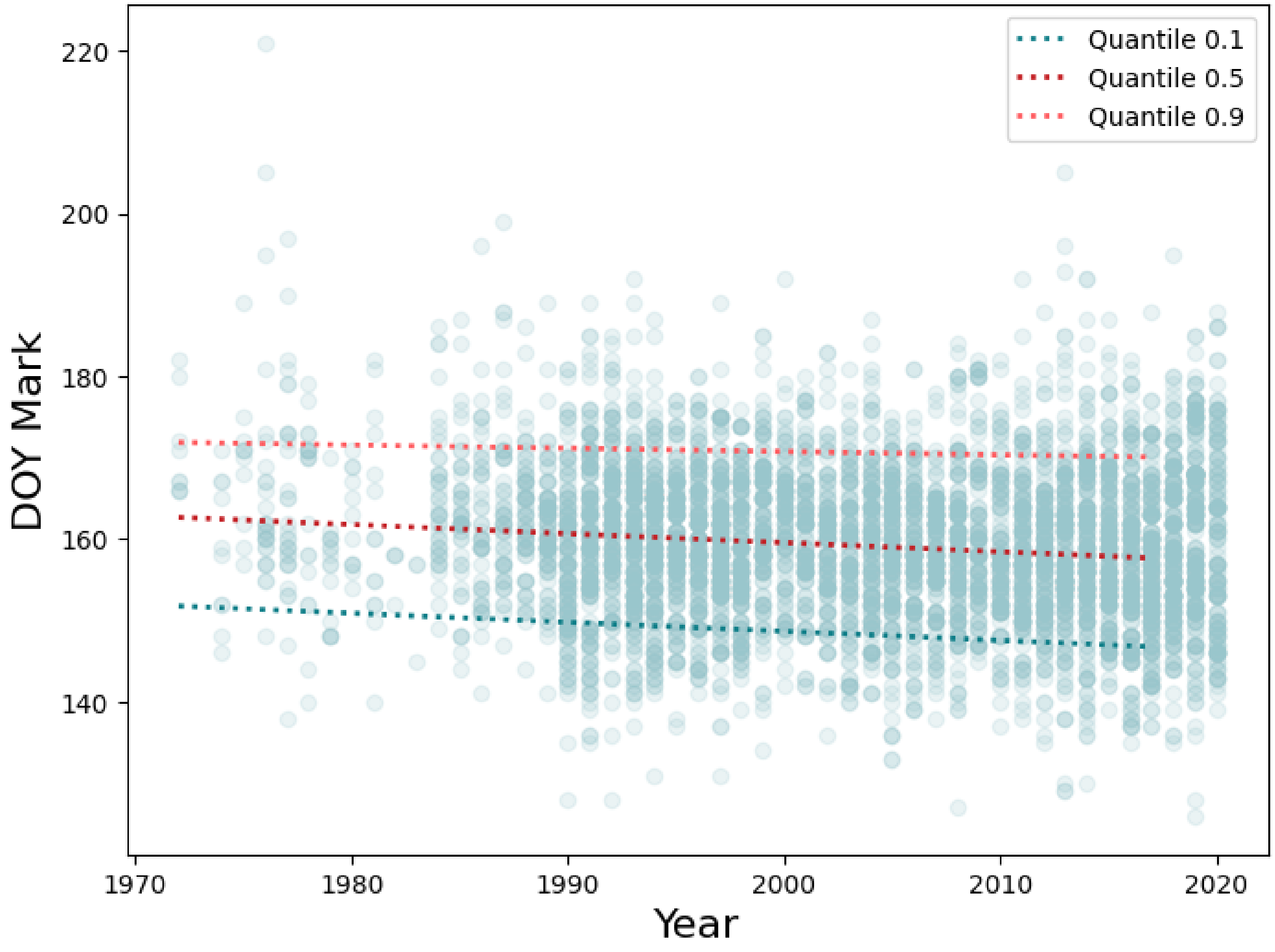

Switzerland : Swiss Engadin

	quantile	Intercept	Slope	p-value
0	0.1	359.965549	-0.103448	0.000053
1	0.5	174.678323	-0.005382	0.813661
2	0.9	-109.857205	0.142857	0.000519

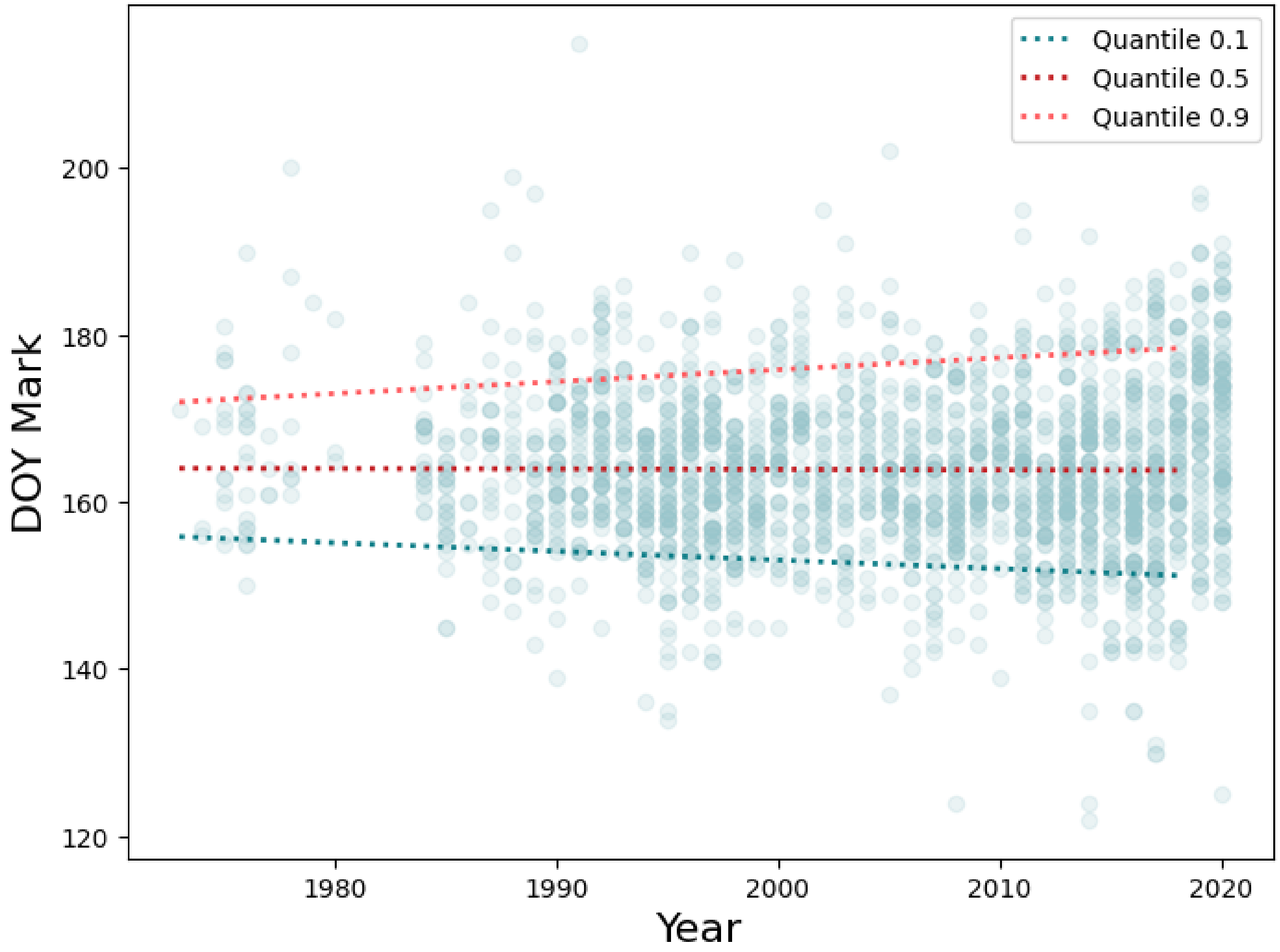

Switzerland : Swiss South Alps

	quantile	Intercept	Slope	p-value
0	0.1	742.353026	-0.294118	1.845027e-14
1	0.5	734.142908	-0.285714	2.619497e-12
2	0.9	807.353126	-0.317249	8.541478e-06

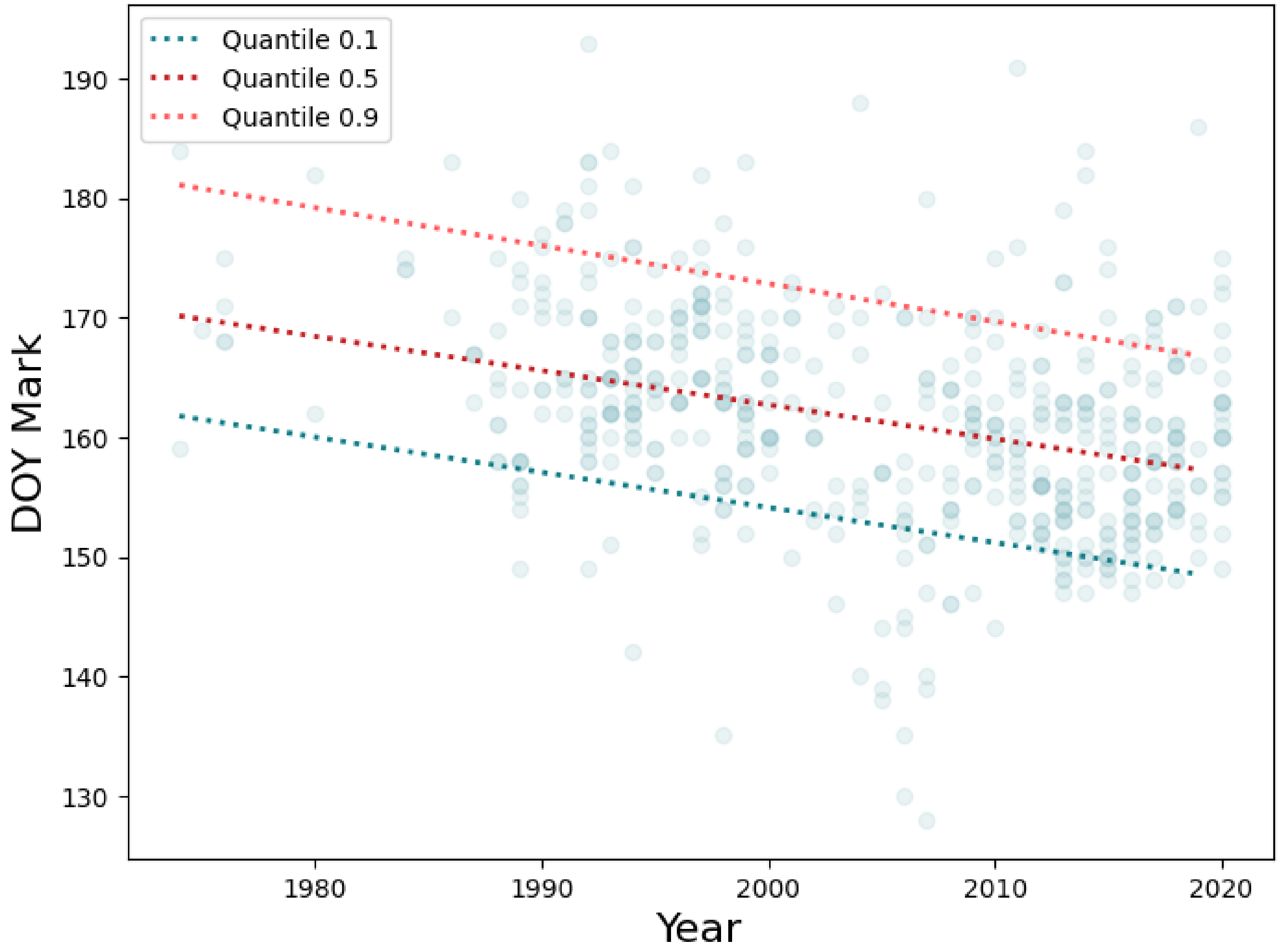

Switzerland : Swiss Western Central Alps

	quantile	Intercept	Slope	p-value
0	0.1	482.000020	-0.166667	0.208493
1	0.5	410.750357	-0.125000	0.279994
2	0.9	-413.726054	0.294421	0.130250

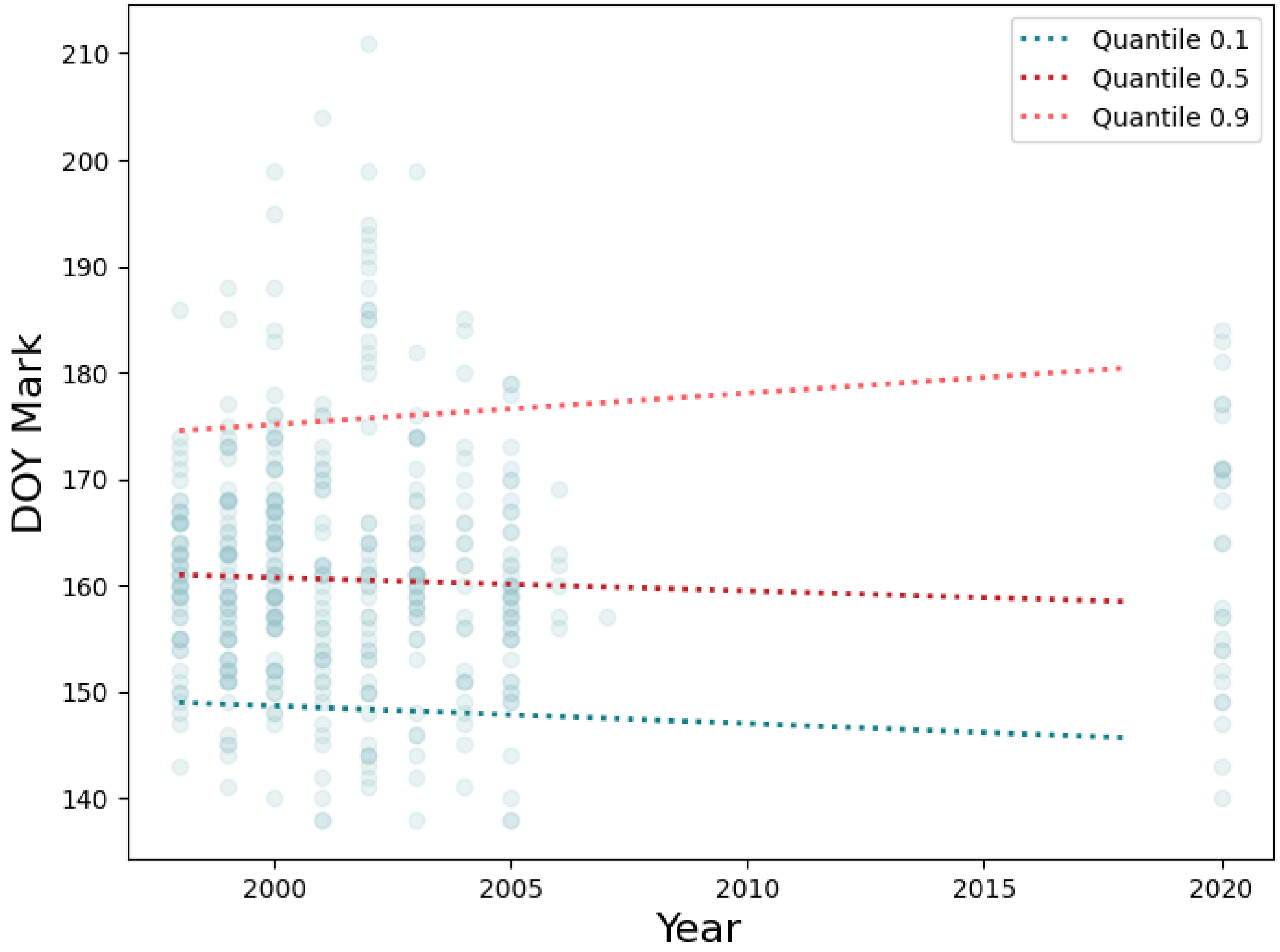

Supplement: Supplementary file 2 — Data S1. Quantile Regression for Switzerland. [file JANE-94-2542-s001.pdf]
